# Systematic mapping of chimera-permissive sites by CRISPR-guided PAM scanning

**DOI:** 10.1016/j.jbc.2026.113627

**Published:** 2026-10-08

**Authors:** Jacinda Pujols, Kyutae D. Lee, Nick J. Kapolka, Samuel Taylor, Bruno Colon, Daniel G. Isom

**Affiliations:** 1 Department of Molecular and Cellular Pharmacology, University of Miami Miller School of Medicine, Miami, Florida, USA; 2 Molecular and Cellular Pharmacology Graduate Program, University of Miami Miller School of Medicine, Miami, Florida, USA; 3 University of Miami Medical Scientist Training Program, University of Miami Miller School of Medicine, Miami, Florida, USA; 4 EvE Bio, LLC, Durham, North Carolina, USA; 5 The Sheila and David Fuente Graduate Program in Cancer Biology, University of Miami Miller School of Medicine, Miami, Florida, USA; 6 Sylvester Comprehensive Cancer Center, University of Miami Miller School of Medicine, Miami, Florida, USA; 7 Frost Institute for Data Science and Computing, Coral Gables, Florida, USA

**Keywords:** CRISPR/Cas9, bioinformatics, synthetic biology, bi-functional chimera, biosensor design, protein engineering, in-cell biophysical protection, signal transduction, yeast, GPCRs, viral protease surveillance, Hepatitis C NS3/4a protease

## Abstract

Protein chimeras enable new biological functions by combining otherwise independent protein elements, but identifying insertion sites that preserve host protein function remains largely empirical. Here, we introduce CRISPR-guided protospacer adjacent motif (PAM) scanning, a scalable strategy for mapping chimera-permissive sites directly in living cells. PAM scanning uses CRISPR-addressable positions across coding sequences to systematically install functional payloads and measure insertion tolerance. In yeast, PAM accessibility provided near-comprehensive coverage across proteins of diverse size and function. We first applied this approach across an entire G protein-coupled receptor (GPCR) signaling pathway, generating 91 chimeras containing inserted protease recognition sequences to map insertion tolerance and site-specific biophysical protection from a protease probe by surrounding protein structure and binding partners. Cognate protease cleavage revealed how this protection varied with protease inhibition and size. We then used PAM scanning to identify bifunctional GPCR chimeras that retained receptor signaling while supporting fluorescent or luminescent reporters. Screening human adenosine A2A and melatonin MTNR1A receptors in yeast identified functional mNeonGreen and NanoLuc insertion sites that retained ligand-dependent signaling following translation to human cells. Together, these studies establish PAM scanning as a general framework for mapping insertional tolerance and biophysical protection in living cells, accelerating functional chimera engineering, and experimentally validating generative chimera design.

Protein chimeras are single polypeptides assembled from domains or sequence elements derived from distinct proteins. In nature, exon shuffling, gene fusion, recombination, and chromosomal rearrangement can recombine pre-existing modules to generate new regulatory, catalytic, and signaling functions, thereby accelerating evolutionary innovation. The same processes can also drive disease. Recurrent oncogenic fusion proteins such as BCR-ABL1, PML-RARA, and EML4-ALK rewire signaling or transcriptional control to promote malignant transformation (1). Natural chimeras, therefore, illustrate both the creative and the pathological consequences of domain recombination, a principle increasingly exploited through protein engineering.

Engineered protein chimeras have become indispensable tools for converting otherwise inaccessible molecular and cellular events into measurable signals. Fusion-based designs, including early GFP and luciferase reporters, transformed cell biology by enabling direct visualization of protein localization, abundance, and dynamics in living systems 2, 3, 4. Insertion-based designs extended this principle, yielding biosensors such as GCaMP and reporters for voltage, cAMP, and kinase activity 5, 6, 7. Chimeric G protein–coupled receptor (GPCR) sensors, generated by inserting fluorescent domains into intracellular loops, further enabled real-time measurement of ligand binding, receptor activation, and effector recruitment 8, 9, 10, 11, 12, 13, 14. Together, these studies established that strategically placed insertions can serve as quantifiable readouts of changes in protein conformation, function, or location.

The modularity of protein chimeras has also enabled major therapeutic advances. Approved biologics such as etanercept, aflibercept, and abatacept combine receptor or regulatory domains with immunoglobulin Fc regions to improve stability, half-life, and efficacy 15, 16. Chimeric monoclonal antibodies, such as rituximab, similarly exploit modular architectures to combine antigen specificity with immune effector function and have transformed the treatment of lymphoma and autoimmune diseases 17, 18. More recently, chimeric antigen receptors used in CAR-T therapies redirect immune cells against cancer, with transformative clinical benefits 19, 20, 21. These examples underscore the therapeutic power of recombining protein modules with defined functions.

Despite their utility, functional protein chimeras remain challenging to design because protein engineering is highly context-dependent. Terminal fusion proteins are often more tractable, as appended domains may spare the host fold. By contrast, insertion chimeras, in which one domain is embedded within another, face stricter constraints to preserve folding, allostery, and native activity while enabling productive interdomain communication. Permissive insertion sites are often sparse and remain difficult to predict from sequence, structure, or computational models alone. Therefore, defining such sites remains a bottleneck in biosensor engineering, synthetic biology, and therapeutic protein design.

Here, we develop a systematic framework for mapping chimera-permissive sites using Clustered Regularly Interspaced Short Palindromic Repeats (CRISPR)-guided protospacer adjacent motif (PAM) scanning. By leveraging programmable genome engineering to create dense insertion opportunities across coding sequences, we identify positions that tolerate domain integration in living cells across diverse protein classes and signaling systems. We apply this approach to define chimeric tolerance *in vivo* across a G protein–coupled receptor (GPCR) signaling pathway, map the in-cell biophysical accessibility of peptide insertions, and identify permissive sites in human GPCRs for biosensor-like readouts. More broadly, this framework enables large-scale functional interrogation of insertional tolerance, reveals principles governing permissive protein architectures, and provides a generalizable approach to accelerate the design of next-generation chimeric proteins.

## Results

### Leveraging high-throughput CRISPR in yeast to explore chimera space

To systematically map chimera-permissive insertion sites in living cells, we sought a platform that combined precise genome editing with scalable functional screening. CRISPR enables targeted sequence diversification and efficient editing at endogenous loci 22, 23, 24, 25. These advantages are especially pronounced in *Saccharomyces cerevisiae* (hereafter yeast), where robust homologous recombination, facile genetics, and scalability enable high-throughput engineering, and where strains can be extensively humanized to reconstitute mammalian signaling pathways and interrogate human proteins 26, 27, 28, 29, 30, 31. We therefore developed a yeast-based CRISPR platform to define insertional tolerance at scale and establish chimeric, genetically encodable protein tools translatable to human, mouse, and other experimentally relevant model systems.

CRISPR targeting relies on a Cas9 nuclease, directed by a single-guide RNA, to create a predictable double-strand break in complementary genomic DNA adjacent to a PAM site (Fig. 1*A*). Because PAM sites are distributed throughout coding sequences, they provide a programmable set of genomic entry points for systematic insertional scanning. We leveraged this constraint, using PAM availability, to nominate codon-resolved positions for domain integration across target genes.

**Figure 1:**
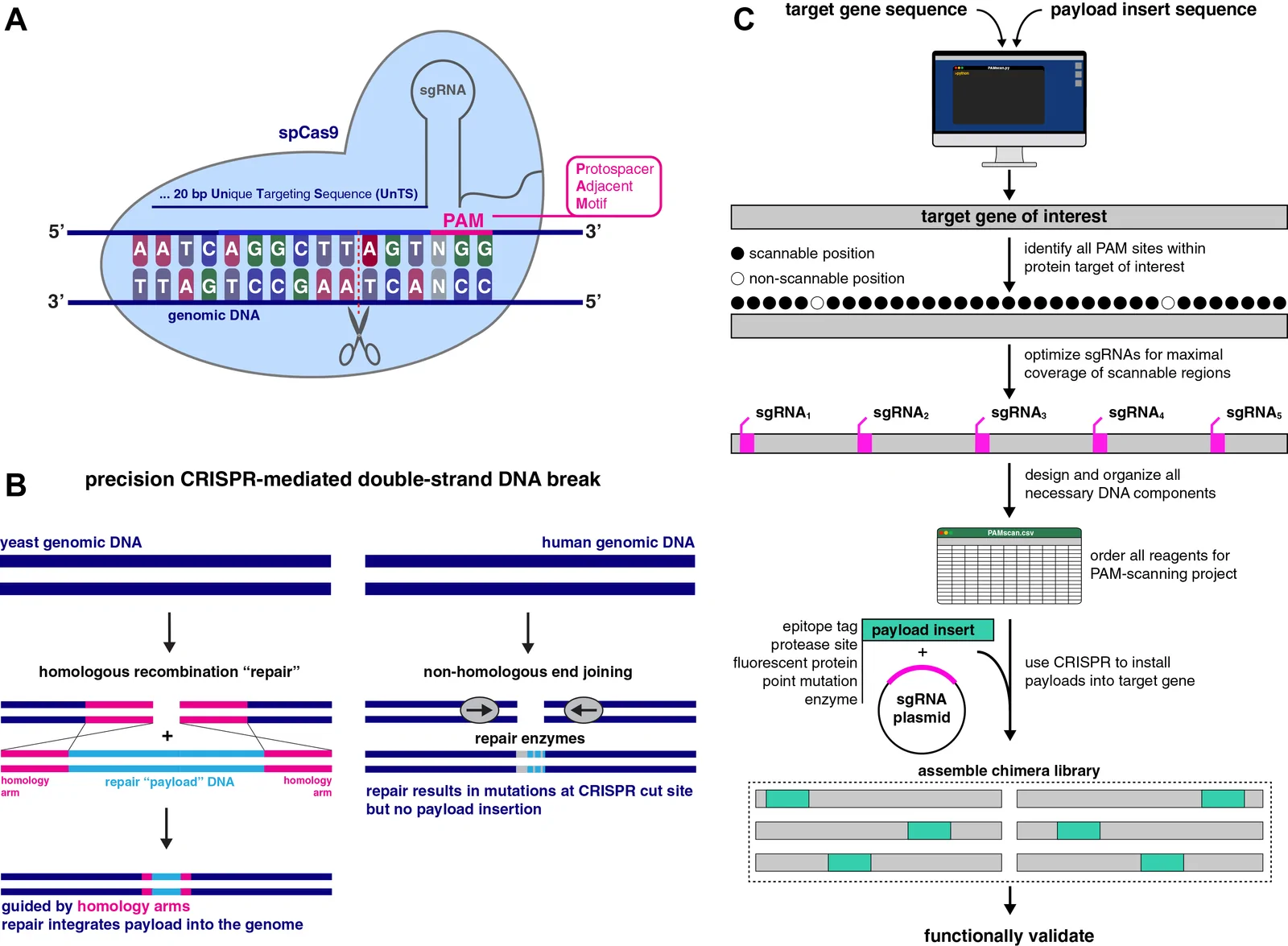
**Principles and workflow of CRISPR-directed PAM-scanning.***A*, SpCas9 recognizes target DNA through a complementary 20-bp single-guide RNA (sgRNA) and a protospacer adjacent motif (PAM; 5′-NGG-3′), with double-strand cleavage occurring three base pairs upstream of the PAM. *B*, schematic of repair outcomes following CRISPR-induced double-strand breaks in yeast and human cells. In yeast (*left*), homologous recombination repairs breaks using a donor template with homology arms (*pink*), enabling precise integration of payload DNA (*dark blue*). In human cells (*right*), repair proceeds predominantly through non-homologous end joining, typically without payload incorporation. *C*, overview of the PAM-scanning workflow. A computational pipeline takes target and payload sequences as input, identifies PAM sites, maps scannable positions, and optimizes sgRNAs to maximize coverage. BLAST assesses off-target potential, and the pipeline assembles selected sgRNAs with donor templates to generate a complete design file. CRISPR-directed insertion produces libraries of chimeric variants, which are screened for functional outcomes.

This strategy is particularly effective in yeast, which efficiently repairs CRISPR-induced breaks through homologous recombination using exogenous donor templates containing payload sequences flanked by homology arms (Fig. 1*B*). In contrast to mammalian systems, where non-homologous end joining frequently predominates, yeast supports precise, parallel installation of designed inserts at high efficiency 26, 27, 28, 29, 30, 31, 32. We therefore implemented a PAM-scanning workflow that computationally identifies accessible PAM sites and designs guide RNAs and donor templates to generate libraries of insertion chimeras for functional analysis (Fig. 1*C*).

A key question is whether PAM sequence requirements provide sufficient coverage of coding sequences for meaningful insertional scanning. To address this, we quantified accessible codons across proteins examined in this study and a representative set of human proteins spanning diverse structural and functional classes (Fig. 2). Because editing efficiency decreases as the distance between the SpCas9 cleavage site and the targeted codon increases, we applied a 30-bp accessibility rule: a codon was considered accessible if a guide directed cleavage within 30 bp of that position. PAM sites were sufficiently frequent to permit broad, often near-comprehensive, exploration of payload insertion sites, as shown by codon-resolved scannability maps for four components of the yeast mating pathway (Fig. 2*A*). This pathway provides a well-established model of GPCR signaling and serves as the basis for our initial experimental application of PAM scanning (Fig. 3*A*).

**Figure 2:**
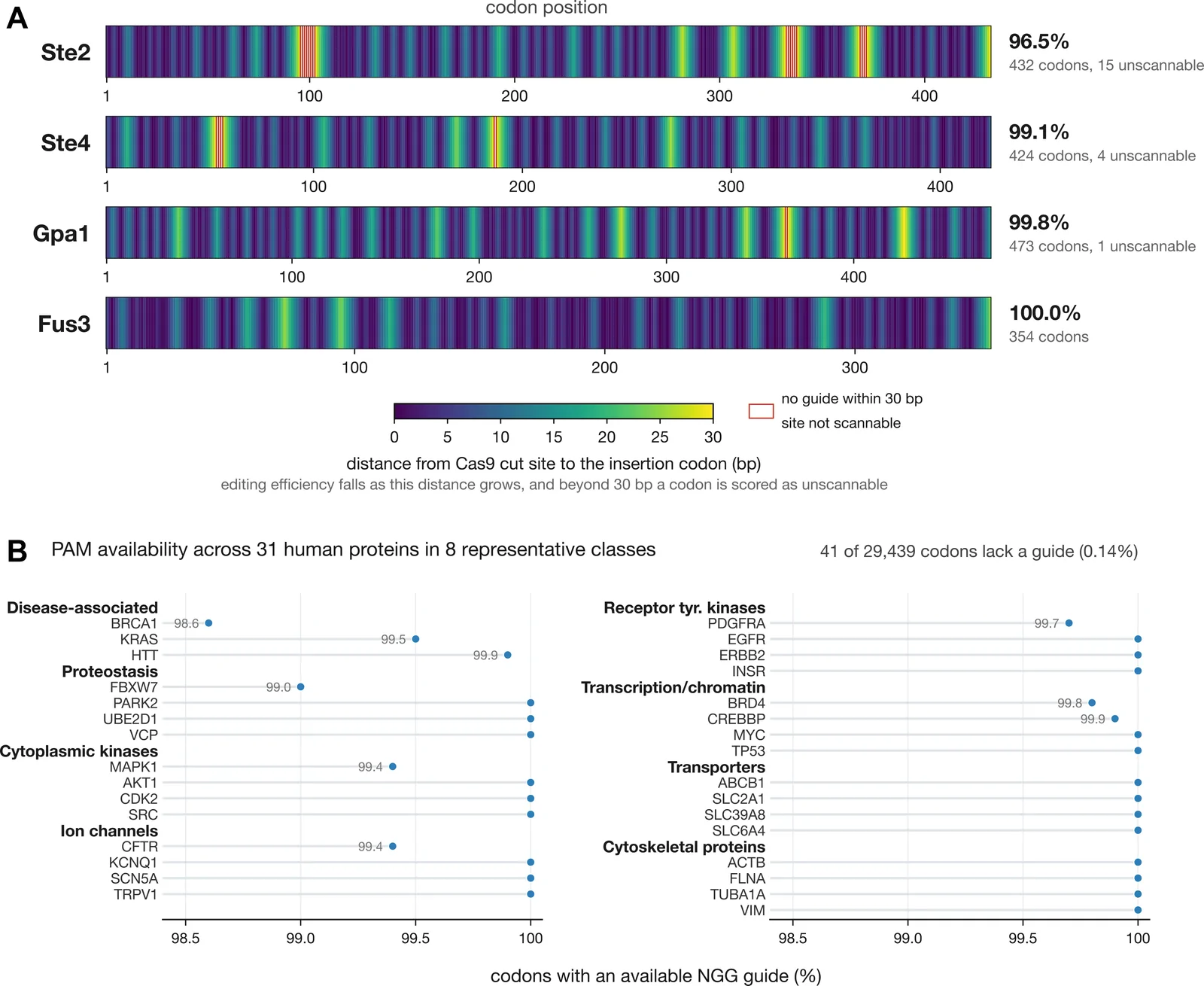
**PAM availability enables near-comprehensive coding-sequence coverage.***A*, codon-resolved scannability of four components of the yeast mating pathway, ordered by increasing coverage. Each cell represents one codon and is colored according to the distance between that codon and the SpCas9 cut site of the closest available guide. Darker cells indicate shorter distances and therefore more favorable positions for editing. Codons lacking a guide that directs cleavage within 30 bp are classified as unscannable under the 30-bp rule and shown in *white* with a *red outline*. Coverage ranges from 96.5% for Ste2 (15 of 432 codons unscannable) to 99.1% for Ste4 (4 of 424), 99.8% for Gpa1 (1 of 473), and 100% for Fus3 (0 of 354). *B*, PAM accessibility across 31 human proteins spanning eight structural and functional classes. Each point represents one protein and indicates the percentage of codons with an NGG guide directing cleavage within 30 bp; values below 100% are shown. Proteins are grouped by class and ordered within each class by coverage. Across all 31 proteins, only 41 of 29,439 codons lacked a qualifying guide (0.14%). Per-protein coverage ranged from 98.6% to 100% (median, 100%), and 22 proteins were scannable at every codon. This analysis was performed computationally; we did not synthesize or experimentally test guides from this panel.

**Figure 3:**
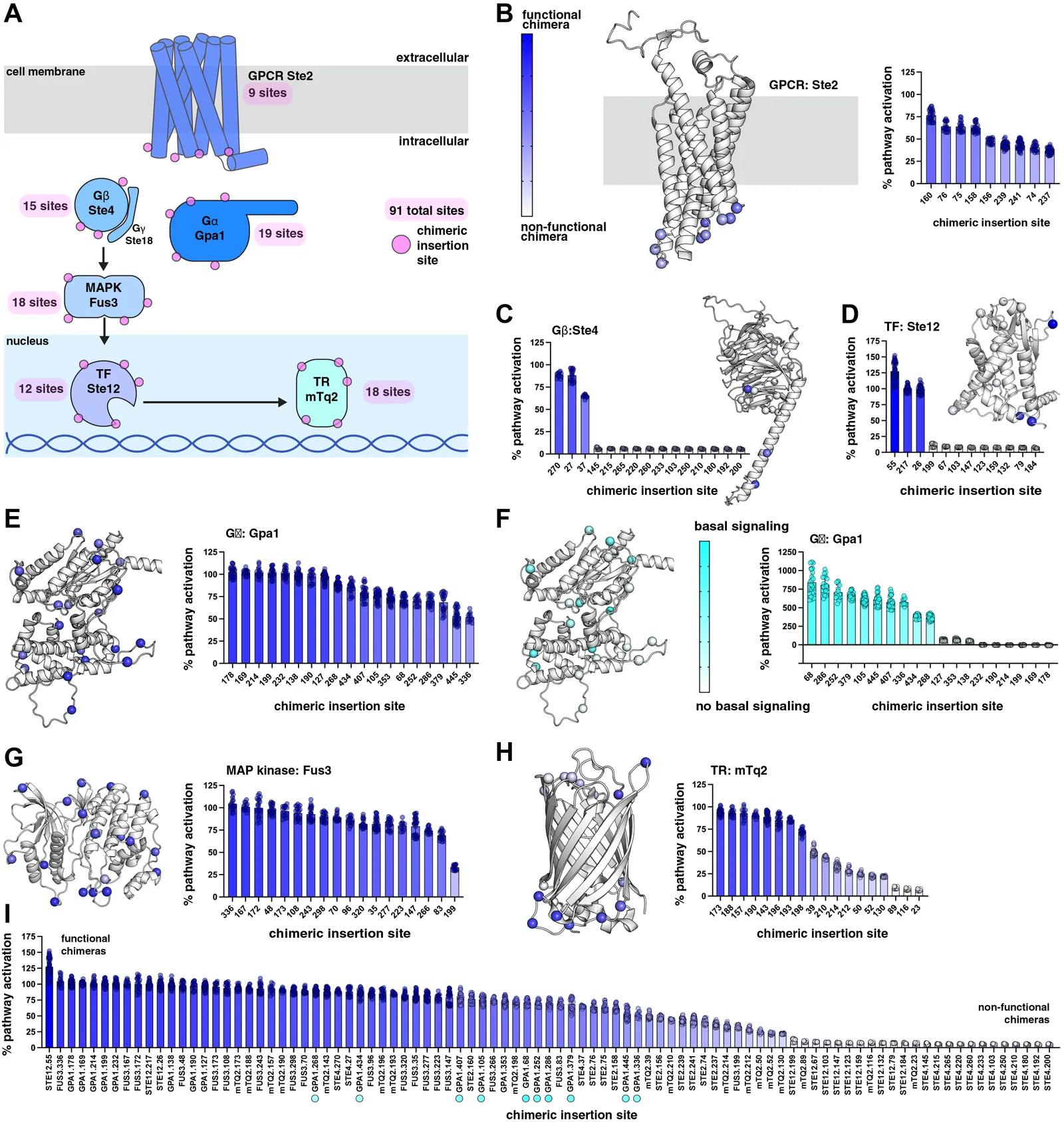
**Global mapping of peptide insertion tolerance across the yeast mating pathway.***A*, schematic of the yeast mating pathway indicating the number of PAM-scanned insertion sites in each signaling component. Chimeras incorporate the HCV NS3/4A protease substrate (EDVVCCSMSY). *B–E, G *and* H*, signaling output of chimeras containing the 10-mer HCV protease substrate. *Spheres* (structural models) and bars (graphs) are color-coded from *dark blue* (functional) to *white* (non-functional). Pathway activation was induced with 10 μM α-factor (18–24 h) and quantified by endpoint fluorescence of the mTq2 transcriptional reporter. *F*, basal signaling activity of Gpa1 (Gα) chimeras measured in the absence of α-factor (18–24 h). *Spheres* and bars are color-coded from *bright cyan* (high basal activity) to *white* (none). *I*, waterfall plot summarizing signaling across all 91 insertion sites. Bar colors follow the key in *panel B*; *cyan circles* indicate non-tolerant Gpa1 chimeras with elevated basal activity. Data represent mean ± s.d. of ≥3 independent biological replicates, each with four technical replicates. Where individual points are plotted, each is a single technical replicate. See Figs. S1, S2, S4, S5, S7–10, and S12–15 for additional data.

We next asked whether this high accessibility extended to human proteins amenable to expression in yeast. We analyzed 31 proteins spanning eight structural and functional classes, including ion channels and transporters, receptor and cytoplasmic kinases, cytoskeletal and proteostasis factors, transcriptional and chromatin regulators, and disease-associated proteins (Fig. 2*B*). Across 29,439 codons, 99.9% were accessible under the 30-bp rule, with coverage for individual proteins ranging from 98.6% to 100% (median 100%). Twenty-two of the 31 proteins were scannable at every codon, and only 41 codons across the entire dataset lacked a usable guide. Coverage remained uniformly high across protein classes and ORF lengths ranging from 148 to 3145 codons. These results indicate that PAM availability places little practical constraint on payload positioning across diverse human proteins amenable to study in yeast.

### Automated design of PAM-defined insertion libraries

To enable systematic mapping of chimera-permissive sites, we developed software that automatically identifies CRISPR-addressable insertion sites and designs corresponding chimeric payloads. The workflow accepts a protein-coding open reading frame along with its flanking untranslated regions. For higher eukaryotic genes, which are frequently interrupted by multiple introns, reducing genomic structure to the spliced open reading frame substantially simplifies scanning and codon-resolved payload design while preserving the encoded protein sequence.

The pipeline then surveys both DNA strands for SpCas9 recognition motifs and identifies all candidate PAM sites. Because spCas9 cleavage can support editing across a local genomic window, each PAM site may access multiple nearby codons, allowing a single cut site to interrogate several potential insertion positions. This establishes a complete editable landscape for each target protein and enables either exhaustive library generation or structure-guided prioritization of informative sites.

For each target position, the software automatically generates the oligonucleotides required to construct guide RNA plasmids and donor templates. For small payloads, the insert sequence can be encoded directly within the primer overhangs. For larger payloads, primers are designed to amplify and assemble pre-existing inserts. This automation converts protein sequences into synthesis-ready reagent sets for arrayed PAM-scanning experiments.

As outlined in Figure 1*C*, the algorithm further incorporates sequence safeguards to preserve desired edits after integration. Where possible, synonymous substitutions eliminate the PAM while maintaining the encoded amino acid sequence, preventing recurrent Cas9 cleavage after payload installation. When direct PAM disruption was not feasible, we introduce synonymous mismatches into the protospacer to reduce guide recognition while preserving host protein-coding potential.

We then filter candidate guides by local BLAST+ analysis to minimize potential off-target alignments in the yeast genome. We then rank remaining positions by the distance between the spCas9 cut site and the codon of interest, and generate flanking primers with universal suffixes compatible with our integration vectors. The final output is a genome-indexed catalog of PAM-scannable codons and corresponding reagents, enabling systematic payload installation across all target proteins. Across proteins examined here, the fraction of PAM-accessible codons was consistently high, supporting broad exploration of insertional tolerance.

### Global mapping of peptide insertion tolerance across the yeast mating pathway

As an initial application of PAM scanning, we systematically explored chimera tolerance and in-cell biophysical protection across a signaling pathway by introducing peptide inserts that confer susceptibility to a cognate protease. We implemented this strategy in the yeast mating pathway (Fig. 3*A*), an insulated GPCR signaling cascade that provides a quantitative readout for protein engineering 26, 27, 28, 29, 30, 31. In this system, the GPCR Ste2 detects extracellular mating pheromone (α-factor) and activates a heterotrimeric G protein complex comprising Gα (Gpa1) and Gβγ (Ste4/Ste18) subunits, with downstream signaling through the MAP kinase Fus3 and transcription factor Ste12 driving expression of the fluorescent reporter mTurquoise2 (mTq2). Using the Hepatitis C virus (HCV) NS3/4A serine protease recognition sequence EDVVCCSMSY as a defined payload, we introduced peptide insertions into pathway components and assessed chimera tolerance *via* α-factor-stimulated mTq2 output.

To achieve pathway-wide coverage, we generated 91 peptide insertion chimeras across the six mating pathway components (Fig. 3). We selected insertion sites from the full PAM-accessible landscape by integrating experimental structures with AlphaFold2 predictions, prioritizing positions distributed across each protein fold with a bias toward solvent-exposed loops and, for transmembrane receptors, intracellular regions outside the plasma membrane. Within the G protein module, we excluded Ste18, the Gγ subunit, due to its small size and obligate scaffolding role in stabilizing the Ste4 β-propeller. We instead treated the heterotrimer as a single structural unit while avoiding protein-protein interfaces. Insertion sites for each component are mapped onto their structures in Figs. S1, S4, S7, S9, S12 and S14.

We then assessed insertion tolerance across the pathway. Of the 91 sites, 57 (63%) retained measurable signaling above baseline, indicating broad tolerance to peptide insertion. All sites in Ste2 and Fus3 supported robust pathway signaling (Fig. 3, *B* and *G*), yet signaling output spanned a continuum across tolerated sites, indicating that insertion position modulates pathway activity rather than simply permitting or abolishing function. Similarly, nearly all sites in the mTq2 transcriptional reporter (15 of 18) were tolerated (Fig. 3*H*). Primary signaling data for every chimera are provided in Figs. S2, S5, S8, S10, S13 and S15.

Of the remaining 34 non-tolerated sites, 24 were mapped exclusively to Ste4, Ste12, and the mTq2 reporter (Fig. 3, *C*, *D* and *H*). Disruption of the β-propeller fold or destabilization of the heterotrimeric complex in Ste4 chimeras led to 12 non-tolerated sites, suggesting reduced permissivity of this fold (Fig. 3*C*). Ste12 insertion positions were guided by a lower-confidence AlphaFold2 model, which limited precision and yielded nine non-tolerant sites (Fig. 3*D*). The final three sites occurred in mTq2 (Fig. 3*H*), where the structurally constrained β-barrel fold is expected to exhibit position-dependent insertion sensitivity.

The remaining 10 intolerant sites mapped to Gpa1 but exhibited a distinct phenotype, reflecting a defining feature of this system. In the yeast mating pathway, the Gβγ subunit drives signaling, whereas in many mammalian GPCR systems, Gα stimulates downstream signaling by producing second messengers. In this context, Gpa1 sequesters Gβγ to suppress basal activity, so disrupting this interaction by peptide insertion can lead to signaling in the absence of an α-factor agonist.

Consistent with this model, insertion chimeras at 10 of 19 Gpa1 sites exhibited substantial basal signaling above baseline (Fig. 3*F*), indicating altered sequestration of Gα-Gβγ. These effects spanned a continuum of basal activity, demonstrating that insertion position differentially modulates the extent of disruption. Despite elevated basal signaling, some chimeras remained responsive to agonist (Fig. 3*E*), indicating that pathway function is retained despite partial loss of regulation at the Gα-Gβγ interface.

Together, these results show that peptide insertions are broadly tolerated across the yeast mating pathway, with 63% of sites retaining measurable signaling (Fig. 3*I*). Tolerance varies by protein: Ste2, Fus3, and mTq2 accommodate insertions at most positions, whereas intolerance clusters in Ste4, Ste12, and Gpa1. Within tolerated sites, insertion position modulates pathway output across a continuum rather than simply preserving or abolishing function. In Gpa1, many insertions disrupt Gα–Gβγ sequestration and induce basal signaling, while remaining responsive to agonist stimulation. These results demonstrate that it is possible to explore chimera space at the pathway scale to identify insertion sites that install new functionality while preserving core protein function.

### Global mapping of in-cell protection across the yeast mating pathway

The concept of protection reflects how structure and macromolecular assembly govern the accessibility of specific protein sites. Given the stable, yet dynamic nature of protein structure, buried or sterically occluded sites are less frequently exposed, whereas flexible or solvent-exposed sites are more accessible. Classical approaches such as hydrogen–deuterium exchange 33, 34, 35, pulse proteolysis (36), and fast quantitative cysteine reactivity 37, 38 can be used to probe and quantify these properties, but are limited to purified proteins.

Having shown that peptide insertions were broadly tolerated across the mating pathway, we expanded our chimera library to address this challenge and measure in-cell protection. Each HCV peptide insertion strain was paired with a companion strain expressing both a chimera and the cognate HCV NS3/4A protease, yielding a total of 182 CRISPR-engineered strains. This matched insertion-protease design enables each engineered site to report local accessibility within its native context and, to our knowledge, establishes the first pathway-scale measurement of site-specific protection in living cells.

As illustrated in Figure 4*A*, proteolytic cleavage can produce two opposing responses. In one case, cleavage reduces or abolishes pathway activity (Fig. 4*A*, left). In the other, cleavage relieves an inhibitory constraint, specifically Gα-mediated sequestration of Gβγ, and causes or elevates basal signaling (Fig. 4*A*, right). Ste2 exemplifies the first behavior, where cleavage of the insertion site disables receptor function and reduces agonist-stimulated output. Gpa1 exemplifies the second behavior, where cleavage disrupts the Gα–Gβγ interaction, thereby releasing Ste4 and elevating basal signaling, an effect suppressed by protease inhibition.

**Figure 4:**
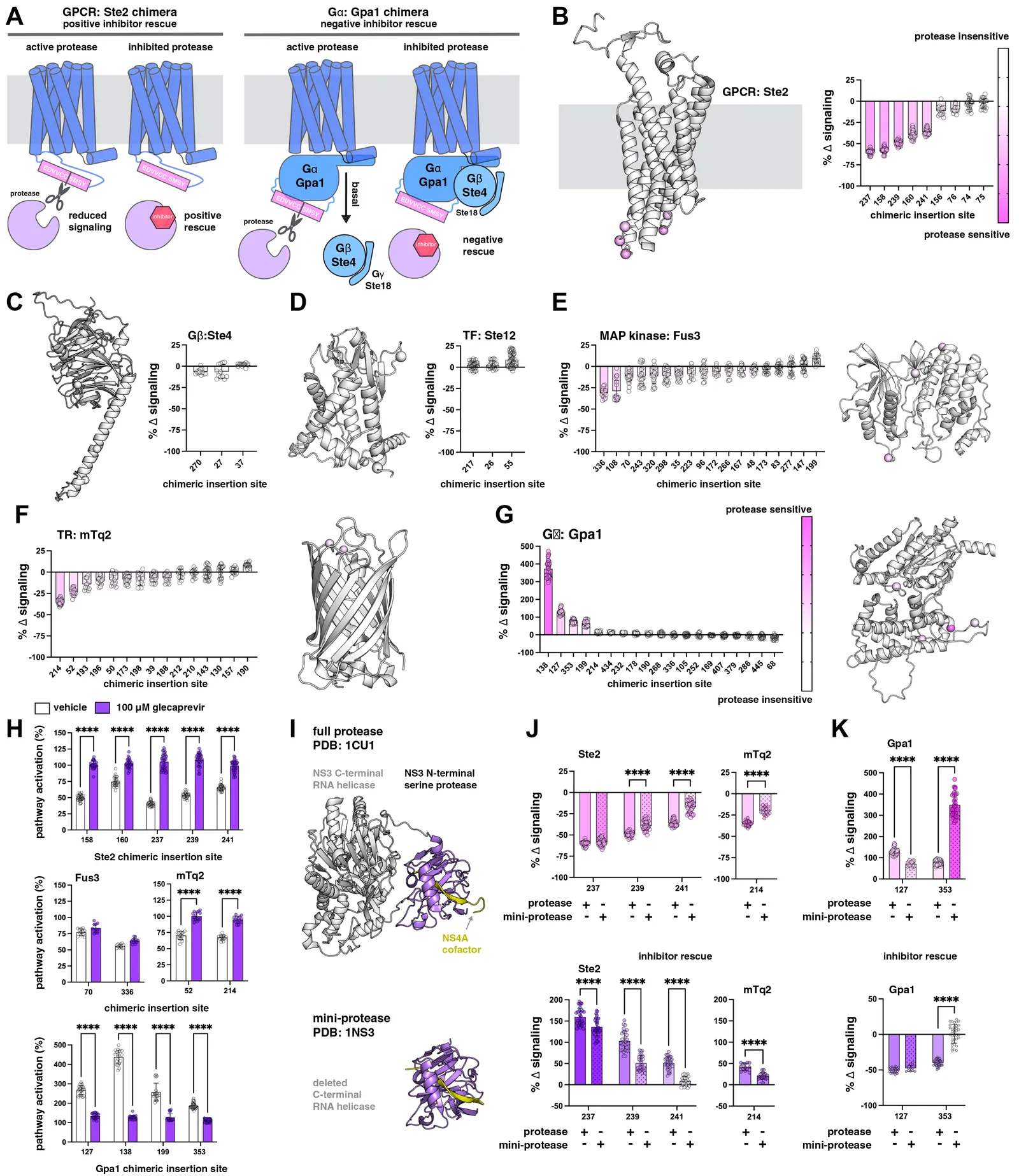
**Global mapping of in-cell protection across the yeast mating pathway.***A*, schematic of protease-dependent modulation of pathway signaling. Cleavage by the HCV NS3/4A serine protease yields two outcomes: loss of α-factor–dependent signaling with restoration upon inhibition (positive rescue, *left*), or induction of signaling upon cleavage with loss upon inhibition (negative rescue, *right*). *B–G*, protease-dependent changes in signaling (±protease) for functional chimeras measured in the presence (*B–F*) or absence (*G*) of α-factor. Spheres (structural models) and bars (graphs) are color-coded from *dark pink* (protease-sensitive) to *white* (insensitive). *H*, inhibitor rescue of protease-sensitive Ste2, Fus3, mTq2, and Gpa1 chimeras treated with glecaprevir (*purple*) or vehicle (*white*). *I*, structural models of full-length (PDB: 1CU1) and truncated mini-protease (PDB: 1NS3) of HCV NS3/4A. The mini-protease comprises the NS3 serine protease domain (*purple*) lacking the RNA helicase domain (*grey*); the NS4A cofactor is shown in *yellow*. *J *and* K*, Comparison of full-length and mini-protease activity. *Top*, percent change in signaling in the presence (*J*) or absence (*K*) of 10 μM α-factor. *Bottom*, inhibitor rescue with 100 μM glecaprevir in the presence (*J*) or absence (*K*) of 10 μM α-factor. Cultures were normalized and incubated for 18 to 24 h. Data represent mean ± s.d. of ≥3 independent biological replicates, each with four technical replicates. Where individual points are plotted, each is a single technical replicate. Statistical analysis was performed using two-way ANOVA with Šídák’s multiple comparisons test or by unpaired *t* test, as specified in the Experimental procedures (∗∗*p* ≤ 0.01, ∗∗∗*p* ≤ 0.001, ∗∗∗∗*p* ≤ 0.0001). See Figs. S3, S6, S11 and S16 for additional data.

To confirm that these responses required proteolytic cleavage, we assayed a protease-sensitive Ste2 chimera (insertion site 237; Fig. 4*B*) against catalytically inactive NS3/4A variants carrying alanine substitutions at either of two catalytic-triad residues (H57A or S139A). Neither inactive variant reproduced the protease-dependent change in signaling (Fig. S17), demonstrating that the response requires catalytic activity of the cognate protease rather than protease expression alone. Because this control preserves the insertion cassette and cleavage sequence while eliminating protease activity, it isolates proteolytic cleavage as the basis of the observed response.

As shown in Figure 4, *B–G*, most of the 57 tolerant chimeras (Fig. 3*I*) were protected from protease cleavage, indicating their peptide inserts are occluded within their native structural or complex contexts. Protection was widespread across components, including Fus3 (16 of 18 sites; Fig. 4*E*) and mTq2 (13 of 15 sites; Fig. 4*F*), as well as all tolerated sites in Ste4 and Ste12 (Fig. 4, *C* and *D*). In contrast, Ste2 showed more limited protection, with 4 of 9 sites protected, consistent with greater accessibility of its intracellular loops (Fig. 4*B*). Similarly, in Gpa1, 4 of 9 sites that did not exhibit substantial basal signaling (Fig. 3*E*) exhibited limited protection (Fig. 4*G*), as protease cleavage at these positions caused basal signaling. Primary protection and inhibitor-rescue data for each component are provided in Figs. S3, S6, S11 and S16.

Having established that proteolysis alters signaling at less protected sites, we next tried to reverse these effects by treating cells with the clinically approved NS3/4A inhibitor glecaprevir 39, 40 (Fig. 4*H*). In this framework, inhibitor treatment should suppress or prevent cleavage to restore chimera function, yielding either positive (Fig. 4*A*, left) or negative (Fig. 4*A*, right) rescue of pathway output. As shown in Figure 4*H*, glecaprevir restored signaling at all protease-sensitive sites in Ste2 and mTq2 (positive rescue) and robustly suppressed protease-induced basal signaling by restoring Gpa1 sequestration of Gβγ (negative rescue).

In contrast, Fus3 sites could not be rescued (Fig. 4*H*), indicating a distinct mechanism of protection. Constitutive cleavage of Fus3 is consistent with an EX1-like regime, in which sites are persistently exposed and efficiently cleaved 33, 34, 35, 36, 37, limiting recovery of protein function. In contrast, Ste2, mTq2, and Gpa1 exhibited inhibitor-responsive behavior consistent with an EX2-like regime 33, 34, 35, 36, 37, in which sites dynamically alternate between protected and exposed states and are cleaved intermittently. In this regime, inhibitor treatment restores signaling by shifting the population toward intact protein.

Beyond structural accessibility, probe size is a key determinant of protection, with smaller probes accessing occluded sites more effectively. To test this *in vivo*, we repeated proteolysis and rescue experiments for select chimeras using a truncated HCV NS3/4A protease (Fig. 4*I*). The N-terminal domain of NS3 encodes the serine protease activity, stabilized by the NS4A cofactor, whereas the C-terminal domain contains a DExH/D-box RNA helicase. Removing this helicase domain generates a smaller mini-protease that retains catalytic activity (41).

Using this mini-protease, we assessed site protection in Ste2 and Gpa1 at select sites. As shown in Figure 4, *J* and *K*, the mini-protease produced modest but consistent additional reductions in signaling at Ste2 insertion sites (239 and 241) and at an mTq2 site (214) compared with the full-length protease (Fig. 4*H*), indicating enhanced access to these recognition sites. Inhibitor treatment restored signaling when the full-length protease was expressed, but rescue was diminished with the mini-protease and was not observed at Ste2 site 241. These results are consistent with increased access shifting these sites from an EX2-like regime with the full-length protease to an EX1-like regime with the mini-protease.

Performing this analysis on select Gpa1 sites (Fig. 4*J*), we observed two distinct outcomes. At site 353, the mini-protease produced greater basal signaling than the full-length protease, and inhibitor treatment suppressed basal signaling only with the full-length enzyme, consistent with enhanced cleavage by the smaller protease. This behavior further indicates that increased accessibility shifts this site from an EX2-like regime with the full-length protease to an EX1-like regime with the mini-protease. In contrast, site 127 showed comparable inhibitor rescue with both protease forms, indicating that cleavage at this position is less sensitive to enzyme size.

Together, these results show that in-cell biophysical protection can be resolved at site resolution across a signaling pathway by integrating proteolysis, inhibition, and probe size. These measurements distinguish EX1-and EX2-like accessibility regimes in living cells, linking structural context to functional outcomes. By leveraging PAM scanning to generate a pathway-wide library of insertion sites, this approach enables systematic interrogation of protection in native protein and complex environments. These findings extend principles established *in vitro* to living cells and provide a generalizable strategy for incorporating peptide-recognition sequences into proteins for tool development, custom enzyme engineering, and monitoring viral protease evolution and escape.

### Engineering bi-functional yeast GPCR Ste2 chimeras

For our next demonstration of PAM scanning as a generalizable engineering strategy, we engineered bifunctional GPCR chimeras that incorporate reporter proteins while retaining receptor signaling. As shown in Figure 5*A*, using the yeast GPCR Ste2 as a model, we inserted fluorescent (mNeonGreen) and luminescent (NanoLuc) reporters at 10 positions distributed across the N-terminus and extracellular loop 1 (ECL1). Of the 10 insertion sites tested, three produced bi-functional chimeras that mapped to positions within ECL1, indicating a localized region of structural permissiveness. Focusing on these bi-functional chimeras, we found that signaling output was reduced relative to wild-type Ste2, whereas reporter protein function was robust and comparable across all permissive insertion sites.

**Figure 5:**
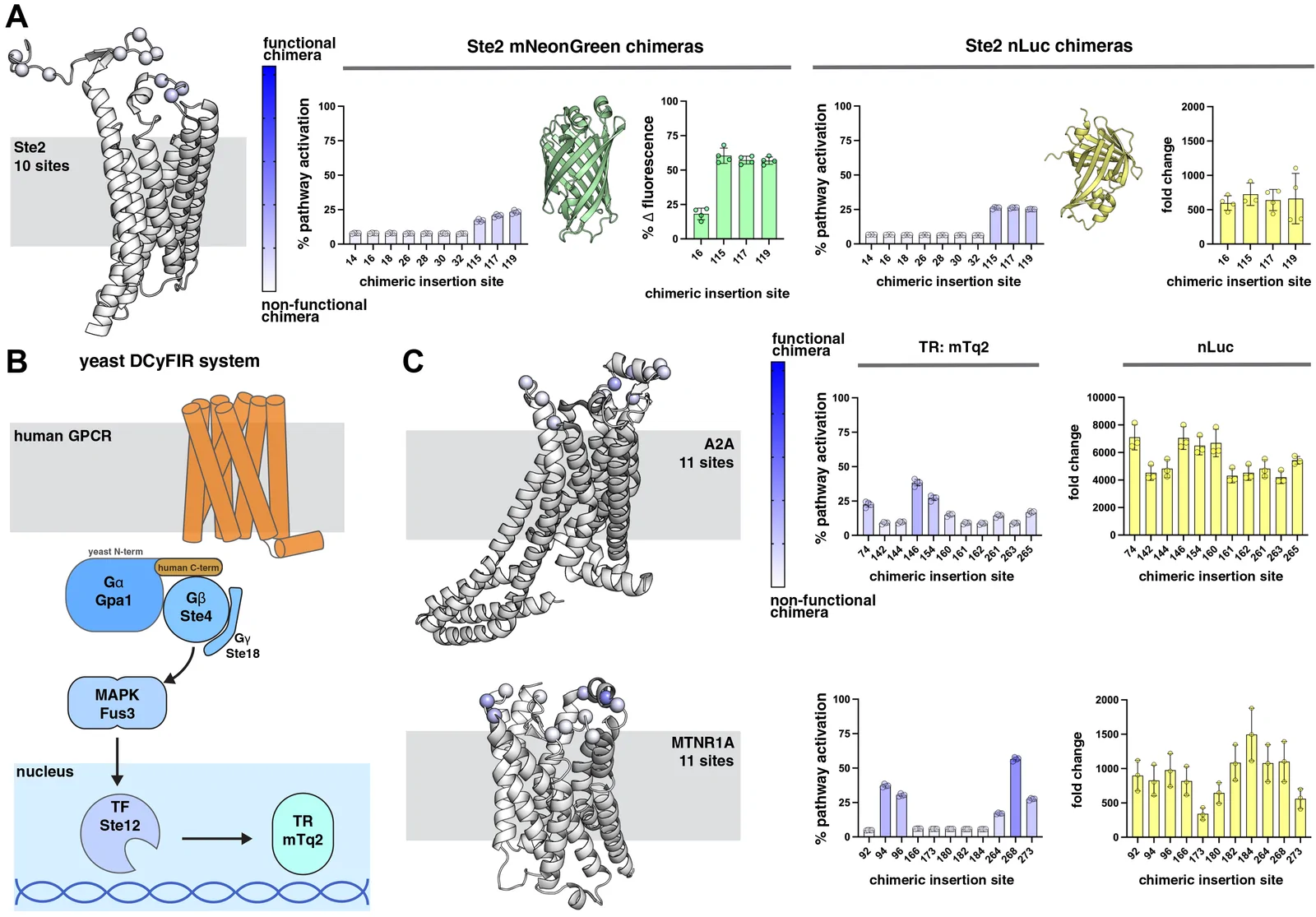
**Engineering bi-functional GPCR chimeras.***A*, functional screen of yeast GPCR chimeras containing inserted mNeonGreen (*left*) or NanoLuc luciferase (nLuc; *right*) reporters. *Left*, Ste2 structure with its 10 scanned insertion sites shown as *spheres* and color-coded from *dark blue* (functional) to *white* (non-functional). Structures of the mNeonGreen and nLuc reporters are shown beside their respective screens. *B*, schematic of the DCyFIR platform adapted for human GPCR signaling in yeast. A chimeric Gα subunit couples human GPCR activation to the yeast pathway by replacing the five C-terminal residues of yeast Gα with those from human Gα (here, Gαi). We measure pathway activation by endpoint fluorescence of the mTq2 transcriptional reporter. *C*, functional screen of human A2A and MTNR1A receptor chimeras containing nLuc inserts. *Left*, A2A and MTNR1A structures, each with 11 scanned insertion sites shown as spheres and color-coded as in *panel A*. Signaling was stimulated with 1 mM adenosine (A2A) or melatonin (MTNR1A). Reporter output (mTq2 fluorescence or nLuc luminescence) and pathway activation were measured in parallel from separate aliquots after 18 to 24 h incubation in the absence of ligand unless otherwise indicated. Data are reported relative to WT as mean ± s.d. (n ≥ 3 biological replicates). Where individual points are plotted, each is a single biological replicate. See Experimental procedures for details.

For non-permissive insertion sites, the absence of signaling did not necessarily reflect loss of Ste2 folding or reporter function. To confirm this distinction, we further investigated the insertion site 16 chimera as a representative example. As shown in Figure 5*A*, although this chimera failed to signal, it retained detectable, albeit reduced, mNeonGreen fluorescence and NanoLuc luminescence levels comparable to bi-functional chimeras. These observations indicate that the site 16 chimera is folded and can support reporter protein function despite its signaling defect. Because inserting one protein sequence into another is expected to substantially perturb local structure, this outcome is likely common across candidate insertion sites, underscoring the need for PAM scanning to systematically identify positions that support true bi-functional chimera engineering.

### Engineering bifunctional human GPCR chimeras

Having demonstrated proof of concept using Ste2, we next turned to the translational engineering of bi-functional human GPCRs. To this end, we employed our previously established DCyFIR platform 26, 27, 28, 29, 30, 31, a yeast-based system for functional interrogation of human GPCR signaling. As illustrated in Figure 5*B*, human GPCRs are expressed in yeast and coupled to a re-engineered mating pathway in which receptor activation engages a chimeric Gα protein (Gpa1) composed of a human C-terminus fused to a yeast N-terminus, enabling productive coupling of human GPCRs to the endogenous yeast signaling machinery. Signal propagation proceeds through the yeast Gβγ subunit Ste4 and the MAP kinase Fus3, culminating in activation of the transcription factor Ste12 and induction of a fluorescent reporter (mTq2). This system enables quantification of human GPCR signaling and provides a robust framework for extending PAM scanning to bi-functional human GPCR engineering.

Using the DCyFIR platform, we applied PAM scanning to two representative human GPCRs, the adenosine A2A receptor (A2A) and the melatonin receptor MTNR1A. As part of the DCyFIR workflow, we CRISPR-integrated each receptor into the genome prior to scanning. We then performed PAM scanning by inserting NanoLuc at 11 extracellular positions (Fig. 5*C*, left) and quantified mTq2 signal output alongside NanoLuc luminescence (Fig. 5*C*, right). Notably, all A2A chimeras retained measurable signaling, albeit with varying magnitudes, indicating broad tolerance for extracellular insertions in this receptor. In contrast, only five MTNR1A chimeras preserved detectable signaling, highlighting more stringent structural constraints. Across both receptors, reporter activity was robust at most insertion sites, even when signaling was compromised, indicating that loss of pathway activation more often reflects disrupted receptor coupling than failed chimera folding or stability.

Building on these yeast-based screening results, we advanced bifunctional chimeras identified by DCyFIR to validation in HEK293T cells using a cAMP-based GloSensor assay (Fig. 6*A*). This strategy leverages DCyFIR for rapid, scalable identification of insertion sites that preserve both receptor signaling and reporter function before evaluation in a human cellular context. In the GloSensor assay, intracellular cAMP binding promotes assembly of a split firefly luciferase, providing a quantitative readout of GPCR-dependent cAMP regulation. Accordingly, activation of Gs-coupled receptors increases luminescence, whereas activation of Gi-coupled receptors decreases luminescence.

**Figure 6:**
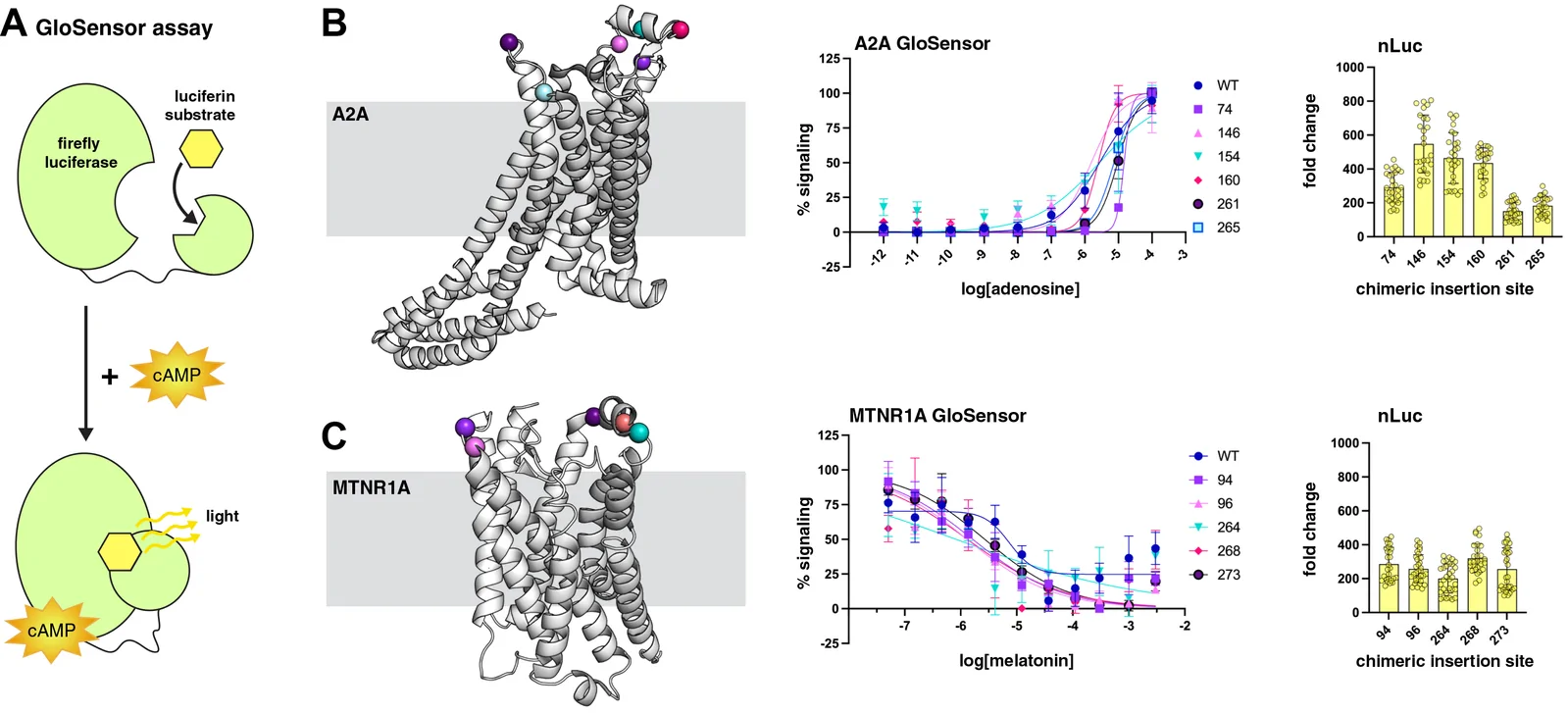
**Validating yeast-screened bifunctional GPCR-reporter chimeras in human cells.***A*, schematic of the GloSensor cAMP assay used to quantify GPCR-stimulated cyclic AMP (cAMP) signaling. *B *and* C*, functional validation of A2A adenosine receptor–nLuc (*B*) and MTNR1A melatonin receptor–nLuc (*C*) chimeras in HEK293T cells. *Left*, structural models with nLuc insertion sites indicated. *Middle*, dose–response curves assessing GPCR signaling. *Right*, endpoint nLuc luminescence confirming reporter function. Insertion sites (*left*) are color-coded to match the corresponding datasets (*middle* and *right*). Data represent mean ± s.d. The dose–response curves (*middle*) are the average of three biological replicates. The nLuc data (*right*) are from ≥3 biological replicates, each with four technical replicates, and each plotted point is a single technical replicate.

HEK293T cells provide a well-established heterologous system for GPCR pharmacology and a standardized background for determining whether chimeras identified in yeast retain bi-functional activity in human cells. Although cellular context may influence quantitative features such as expression, trafficking, and basal signaling, validation in this widely used system allows evaluation of the signaling properties of the engineered receptors within an extensively characterized framework.

Focusing on the subset of A2A and MTNR1A chimeras identified as bi-functional by DCyFIR, we used the GloSensor assay to confirm bi-functional activity in human cells. As shown in Figure 6*B* for the Gs-coupled A2A receptor, each bi-functional chimera exhibited a positive, adenosine-dependent dose–response curve comparable to wild-type A2A, indicating preservation of ligand-dependent activation and coupling to cAMP production. In contrast, as shown in Figure 6*C* for the Gi-coupled MTNR1A receptor, each bi-functional chimera exhibited a negative, melatonin-dependent dose–response curve, again similar to wild-type MTNR1A, consistent with ligand-dependent inhibition of cAMP formation. Across both receptor sets, reporter activity remained robust and readily detectable, confirming maintenance of bi-functional behavior following translation from yeast to human cells.

## Discussion

PAM scanning enables systematic exploration of chimera-permissive sites at a scale difficult to achieve with conventional protein engineering approaches. Because any gene expressible in yeast can be subjected to CRISPR-directed insertion, this framework is broadly applicable to proteins from across all domains of life. DNA-level engineering enables rapid, parallel generation of insertion variants with minimal cost and labor compared with iterative protein-level design. Although we used SpCas9 here, the approach is readily extendable to alternative CRISPR systems with distinct PAM requirements, further expanding the accessible sequence space for insertional scanning. In this way, PAM availability is a programmable feature that defines a tunable landscape for systematic chimera design.

Mapping permissive insertion sites in living cells enables a wide range of applications. Insertion-based reporters and biosensors can be rapidly developed by placing functional domains at sites that preserve host protein activity while reporting conformational or signaling changes. This approach is particularly well-suited for pharmacologically important targets such as GPCRs, where insertion into intracellular or extracellular loops enables real-time measurement of receptor activation or localization and can serve as fiducial markers to facilitate cryoEM studies. More broadly, systematically identifying permissive sites may accelerate the development of engineered proteins for research and therapeutic applications by reducing the need for trial-and-error design.

By combining insertional scanning with site-specific proteolysis, we identified chimeras that remain functional yet are responsive to protease cleavage, enabling reversible control of protein activity in living cells. Protease inhibition restores signaling at these sites, creating a tunable system in which cleavage and rescue modulate function. This creates two immediate applications. First, using optimized insertion sites, pooled experiments can monitor protease activity and inhibition, enabling rapid assessment of the effectiveness of existing inhibitors against naturally occurring or engineered viral protease variants. Second, this system can screen libraries of peptide recognition motifs against a given protease or, conversely, evolve proteases with desired specificity against defined peptide sequences.

Insertion tolerance reflects at least two interacting contributions. Payload size imposes a general constraint, with smaller payloads typically accommodated more readily than larger ones. Payload topology may also be important: proteins with nearby N- and C-termini are naturally suited for insertion, whereas those with distant termini may require long linkers that disrupt the host protein, the payload, or both. Site-specific permissiveness is governed largely by local structural context and may therefore not be preserved across payloads of different sizes, folds, and terminal geometries. Because these combined effects cannot yet be reliably predicted from sequence or structure alone, systematic experimental mapping remains the most practical route to identifying permissive sites. A natural extension of PAM scanning will be to compare payloads with varying sizes, structures, and terminal geometries across matched sets of insertion sites.

Finally, advances in sequence- and structure-based AI models have outpaced the availability of scalable experimental systems for testing their predictions. PAM scanning provides a direct route to close this gap by enabling computational design of insertion libraries and rapid experimental validation in living cells. While current models can estimate structural features, they remain limited in predicting insertion tolerance and functional outcomes. By enabling systematic, site-resolved measurements of insertion tolerance and accessibility, PAM scanning provides ground truth for benchmarking and refining predictive models, enabling iterative cycles of prediction and validation. In this way, PAM scanning couples design and experiment, establishing a scalable framework for data-driven discovery and predictive chimera engineering.

## Experimental procedures

### PAM-scanning chimera design and validation

#### Computational pipeline and software implementation

We performed insertional scanning using a custom Python-based workflow, developed to identify PAM-accessible positions and design synonymous silencing edits across open reading frames (ORFs). The software comprises three integrated modules: (1) a Tkinter-based graphical interface for input specification, (2) a pipeline controller that orchestrates file processing, PAM detection, and BLAST filtering, and (3) a core library implementing codon-level scanning, synonymous mutagenesis, and primer generation. The same pipeline is available as a command-line program that takes these inputs as flags, a tab-separated manifest of many ORFs, or a folder of ORFs discovered by filename convention so that you can script and repeat a scan. A companion command, pam-scan-fetch-cds, retrieves a coding sequence for a UniProt accession, which we used to obtain the human ORFs analyzed here. Both are installed with the package, available from Bioconda and from the repository listed under Data Availability.

The software accepts two required inputs: (*i*) the ORF sequence, from the ATG start codon through the stop codon, and (*ii*) the flanking genomic sequence that surrounds it. The software accepts the flanking sequence either as separate 5′ and 3′ FASTA files or as a single FASTA file containing the ORF and its flanks; in that case, it locates the ORF by exact match and takes the flanks from each side. We assemble the ORF and its flanks internally into an extended ORF + sequence, which allows scanning to reach insertion positions at the start and end of the coding sequence. The flanking sequence may be any length, provided it supports the homology arms used for primer construction; approximately 60 nucleotides are recommended, and shorter arms remain workable. Off-target evaluation uses a host genome FASTA file, which defaults to a bundled *S. cerevisiae* BY4741 assembly and may be replaced with another yeast species, strain, or variant. The software automatically builds a local BLAST+ database from that genome on first use and caches it for subsequent runs, so you don't need to supply a database. Two further inputs are optional: a codon-usage table, which defaults to a bundled yeast table, and a codon-selection sheet listing specific residues for targeted scanning, which may instead be chosen graphically from the translated ORF. The user also provides the gene name and the primer suffixes, along with numerical parameters defining primer length, the maximum allowed distance between the Cas9 cut site and the insertion codon, the codon-sampling stride, and the off-target thresholds.

When run, the program reads the input FASTA sequences and builds a codon index across the ORF. Using a codon-usage table derived from the host organism, the algorithm maps each amino acid to its synonymous codons. The software then scans the extended ORF+ sequence on both strands for Cas9 recognition motifs (NGG and CCN), identifying PAM sites, associated guide sequences, and predicted Cas9 cut positions.

#### Synonymous PAM and guide silencing

To prevent re-cutting of successfully integrated constructs, each PAM site undergoes synonymous “silencing” edits that remove Cas9 recognition without altering the encoded amino acid. We identify codons overlapping or flanking the PAM triplet and introduce synonymous substitutions to eliminate the GG or CC dinucleotide motif. When the PAM lies out of frame or spans two codons, the pipeline evaluates both codons for permissible substitutions based on codon-usage bias.

If direct PAM silencing is not possible, the pipeline performs guide-sequence silencing by introducing synonymous substitutions within the 20-nucleotide protospacer to increase mismatches to the original guide while preserving the protein sequence. This fallback ensures that each site yields a viable “safeguide,” a modified target resistant to Cas9 cutting after chimeric integration.

#### Off-target filtering and guide selection

We screen all candidate guides for potential off-target alignments using local NCBI BLAST+ against the specified host genome. The software parses alignments to identify “PAM inclusions,” defined as partial matches that exceed user-defined identity or alignment-length thresholds. We exclude guides with significant off-target similarity.

We rank remaining safeguide candidates according to two criteria: (1) the distance between the predicted Cas9 cut site and the targeted codon (PAM–cut gap) and (2) the number of PAM inclusions in the host genome. We select the optimal guide per codon based on the minimal gap and the lowest off-target burden. Editing efficiency falls as the distance between the cut site and the targeted codon increases, so we scored a codon as unscannable if no guide cuts within 30 bp. This 30 bp rule defines the scannable fractions reported throughout.

#### Primer generation and library design

For each optimal safeguide, we design oligonucleotide primers flanking the insertion site with user-defined lengths and suffix sequences compatible with the cloning backbone used for Cas9-mediated integration. The pipeline automatically organizes primers into 96- or 384-well plate layouts to facilitate large-scale oligonucleotide ordering.

To assess target coverage, the pipeline computes the fraction of the ORF reachable by valid PAM sites within the maximum allowed cut distance. Output directories include.

• QC/— raw and silenced FASTA files,
• BLAST+/— off-target analysis reports,
• ORDER/— primer order spreadsheets, and
• WARNINGS/— flagged codons with residual PAM inclusions.

#### Chimera construction in yeast

We generated chimeric constructs by CRISPR-mediated homologous recombination in *S. cerevisiae*. For each PAM-scannable position, we used the designed primer pair to amplify and insert a cassette encoding a protease cleavage site or reporter in-frame at the safeguide locus. We selected integration events on defined media and verified them by colony PCR and Sanger sequencing across both junctions. We provide details of CRISPR transformation, payload preparation, and functional validation below.

#### PAM availability across human protein classes

We selected 31 human proteins spanning eight structural and functional classes, excluding GPCRs. We retrieved reference open reading frames from RefSeq. We processed each ORF with the same PAM-scanning pipeline and design rules used for the yeast targets, enumerating, for every codon, whether a guide with an NGG PAM could be positioned to direct insertion at that codon. We scored a codon as scannable when at least one such guide existed, and we report per-protein coverage as the percentage of codons in the open reading frame that were scannable. This analysis is purely computational. We did not synthesize or test any guides from this panel.

### Summary of experimental reagents

Datasets S1–S4 list all guide sequences, primers, plasmids, strains, and reagents used or created in this study.

### Plasmid construction

#### CRISPR PAM-scan plasmid construction

We constructed CRISPR plasmids using the multicopy pML104 X-2 UnTS vector previously developed in our laboratory 26, 28, 29, 31. This plasmid contains a 2-micron origin of replication, encodes Cas9 and a single-guide RNA (sgRNA) scaffold with a 20-base-pair unique targeting sequence (UnTS), and confers URA3 selection in yeast. For each chimera, we replaced the UnTS with a 20-base-pair safeguide sequence targeting a specific codon insertion site. We generated these safeguide sequences using our custom computational pipeline and software implementation described above. We introduced the safeguide into the pML104 X-2 UnTS backbone using a chimera-specific forward primer (annotated as gF in Dataset S1) and a universal reverse primer carrying a 5′ phosphate modification (pML guide UR (5PHOS): 5′-phos-GATCATT TATCTT TCACTG CGGA-3′).

We assembled and circularized the plasmid using the Around-the-World PCR protocol. Each reaction contained 1.25 μl of guide (gF) primer (5 μM), 1.25 μl of pML guide UR (5PHOS) primer (5 μM), 9.0 μl of nuclease-free water, 12.5 μl of Q5 2 × Master Mix (New England Biolabs, M0492L), and 1.0 μl of template plasmid DNA (10 ng/μl). We confirmed the PCR product by agarose gel electrophoresis before adding 1.0 μl of DpnI (NEB, R0176S) to digest methylated template DNA. We incubated the mixture at 37 °C for 1.5 h, then heated to 80 °C for 20 min, and finally held at 4 °C. To ligate the PCR-amplified plasmid, we added 23.0 μl of nuclease-free water, 5 μl of 10 × T4 DNA ligase buffer (NEB, B0202S), and 1.0 μl of T4 DNA ligase (NEB, M0202L), and incubated the reaction overnight at 16 °C. We terminated ligation by heating at 65 °C for 10 min, then transformed the newly assembled plasmids into bacteria as described below.

#### HiFi plasmid construction

For plasmid assembly using HiFi cloning, we PCR-amplified both the plasmid backbone and corresponding insert using HiFi-compatible primers designed for sequence overlap. After PCR, we digested the template DNA with DpnI (NEB, R0176S) to remove the methylated parental plasmid. We prepared the HiFi assembly reaction by combining 14.2 μl of nuclease-free water, 0.5 μl of insert PCR product, 0.3 μl of vector PCR product, and 5 μl of HiFi DNA Assembly Master Mix (New England Biolabs, E2621L) in a single PCR tube, followed by incubation at 50 °C for 15 min. We then introduced the assembled plasmids into *E. coli* (DH5α) by transformation using 2 μl of the final HiFi reaction for amplification and sequence verification, as described in the protocol below.

#### Protease plasmid construction

We obtained the gene encoding the Hepatitis C virus NS3/NS4A protease from Addgene (plasmid #61696) in a pETDuet bacterial expression backbone. Using HiFi DNA assembly, we subcloned the protease coding sequence into the pYE181 yeast vector under the control of the TEF1 promoter and CYC1 terminator using primers pYE MCS F and R (Dataset S1). We did not use the resulting plasmid for direct expression of the protease in yeast, but instead as a storage and amplification vector to provide template DNA for subsequent CRISPR-mediated integration of the TEF1-protease-CYC1 open reading frame into the yeast genome.

#### Mini protease plasmid construction

A truncated version of the Hepatitis C virus NS3/NS4A protease, containing only the N-terminal NS3 serine protease domain and missing the C-terminal RNA helicase domain, was generated using HiFi DNA assembly. The mini protease coding sequence (41) was subcloned into the pYE181 yeast vector under the control of the TEF1 promoter and CYC1 terminator using the primers pYE181.hifi.fwd.ns34a.construct and ns34a.construct.hifi.rvs (Dataset S1). As with the full-length protease plasmid described above, we did not use the resulting construct for direct mini protease expression in yeast; instead, we used it as a storage and amplification vector to provide template DNA for CRISPR-mediated integration of the TEF1–protease–CYC1 open reading frame into the yeast genome.

#### Chimeric GPCR plasmid construction

We generated human GPCR chimera plasmids by PCR-amplifying the chimeric A2A and MTNR1A receptor genes previously integrated into the yeast genome *via* CRISPR editing. We amplified the genes using primer pairs A2A pcDNA3.1 (pos) F/R and MTNR1A pcDNA3.1 (pos) F/R (Dataset S1). We then subcloned the resulting PCR products into the pcDNA3.1(+) expression vector using the HiFi plasmid construction procedure described above, enabling subsequent expression of the chimeric GPCRs in human cell lines.

### CRISPR payload design and preparation

#### CRISPR payload design

We performed yeast CRISPR reactions by transforming a plasmid expressing a single-guide RNA (sgRNA), the Cas9 endonuclease, and a URA3 selectable marker, as described above. Expression of these components generates a precise double-strand break at the genomic locus complementary to the sgRNA target sequence. The payload refers to the repair DNA fragment introduced during this process, consisting of the desired gene or nucleotide sequence flanked by homology arms corresponding to the genomic regions immediately upstream and downstream of the cut site. In PAM-scanning experiments, the payload encoded a peptide or protein sequence, which we inserted into a target gene to generate a chimeric construct. We determined the homology-arm sequences and corresponding primers for payload amplification computationally using the custom pipeline described earlier.

#### CRISPR payload preparation for integrating protease recognition sites

Because the Hepatitis C virus NS3/4A protease recognition sequence (EDVVCCSMSY) is encoded by only 30 nucleotides (5′-GAAGATGTTGTCTGTTGCTCTATGTCATAT-3′), we designed the CRISPR payload as complementary 100-base-pair forward and reverse primers (Dataset S1). To generate double-stranded DNA, we combined equal volumes of each primer (15 μl) with 30 μl of Q5 2 × Master Mix (New England Biolabs) in a PCR tube. We heated the mixture in a thermocycler to 95 °C for 1.5 min, then cooled it gradually at 1 °C per minute to 50 °C to allow primer annealing.

#### CRISPR payload preparation for integrating reporter proteins

For the reporter proteins NanoLuc luciferase (nLuc) and mNeonGreen, we generated CRISPR payloads using a two-step PCR-based process. In the first step, we amplified each reporter gene from its respective storage plasmid or gblock (pNluc-N1, from Lambert Lab; mNeonGreen, IDT-DNA) using primers that appended universal flexible linkers: an N-terminal SGGSGG linker (5′-TCAGGTGGCAGTGGAGGT-3′) and a C-terminal GGSGGS linker (5′- AGACCCGCCGCTACCTCC-3′). In the second step, we re-amplified the reporter constructs using 100-base-pair primers with homology arms specific to the designated chimeric insertion site, generating the final payloads for integration into the yeast genome.

### General molecular biology protocols

#### Bacterial transformation

We prepared chemically competent *E. coli* DH5α cells (a gift from the Dohlman laboratory) and stored them at −80 °C using the Mix & Go! *E. coli* Transformation Kit (Zymo Research, T3002). For transformation, we thawed 50 μl of competent cells on ice for 20 min, mixed them with 2 μl of synthesized plasmid DNA, and incubated them on ice for an additional 5 min. We then spread the transformation mixture onto LB agar plates containing ampicillin (0.01%) and incubated them overnight at 37 °C, with plates inverted during incubation.

#### Plasmid isolation and verification

*We inoculated t*hree individual bacterial colonies into 3 ml of fresh LB broth containing ampicillin (0.01%) in 15 ml Falcon conical tubes and incubated them overnight at 37 °C in a shaking incubator. We isolated plasmid DNA the following day using the EZ BioResearch Miniprep Kit (M1000-250) according to the manufacturer’s protocol. We sequence-verified the purified plasmids by Sanger sequencing (Eurofins Genomics) before downstream applications.

#### Yeast transformation

To prepare a starter culture, we inoculated 3 ml of YPD medium with yeast and incubated it overnight at 30 °C with shaking. The next day, we transferred 100 μl of the overnight culture into 5 ml of fresh YPD and incubated under the same conditions for about 2.5 h, until the culture reached an OD_600_ of 0.6 to 1.2. Harvest cells by centrifugation and discard the supernatant. We sequentially washed the yeast pellet with 5 ml of 1 × TE buffer and 5 ml of LiOAc mix, pelleting after each wash. Following the final wash, we resuspended the pellet in 200 μl of LiOAc mix, sufficient for approximately four transformations.

Each transformation reaction combined 50 μl of chemically prepared yeast cells with 300 ng of plasmid DNA (*e.g.*, CRISPR guide plasmid), 20 μl of repair DNA or donor payload, 350 μl of PEG mix, and 5 μl of salmon sperm DNA (Invitrogen, 15632011) in a sterile 1.5 ml microcentrifuge tube (USA Scientific, 1615-5500). Briefly vortexed samples and incubated them at room temperature for 30 min. After incubation, add 24 μl of 100% DMSO, then heat-shock the cells at 42 °C for 15 min. After heat shock, we pelleted cells by centrifugation (10,000 rpm, 3 min), gently resuspended them in 100 μl of YPD (without vortexing), and plated them onto appropriate selective media (*e.g.*, –Ura plates) for approximately 3 days at 30 °C. Unless otherwise noted, we pelleted all yeast by centrifugation at 3000*g* for 3 min.

#### Yeast strain verification

We inoculated 3 to 12 yeast colonies into individual wells of a 96- well block, each containing 1 ml of –Ura selection medium. We sealed the block with a breathable film (Thomas Scientific, 1198M9), shook it briefly at 1400 rpm for 1 min, and then incubated it without shaking for approximately 2 days at 30 °C. After incubation, we verified chimeric integrations using the genomic DNA (gDNA) extraction and verification protocol described below. We streaked confirmed chimeras onto 5-fluoroorotic acid (5-FOA) plates and incubated them for 2 to 3 days at 30 °C.

From each 5-FOA plate, we selected three colonies and reinoculated them into fresh wells of a 96- well block containing 1 ml of YPD medium per well. We incubated the block overnight at 30 °C, then repeated the gDNA verification protocol to confirm the strain identity. We prepared verified YPD cultures for long-term storage by mixing 300 μl of culture with 130 μl of 50% glycerol (Sigma-Aldrich, G6279-1L) and storing the glycerol stocks at −80 °C.

### Yeast media

#### Yeast extract peptone dextrose media (YPD)

We prepared yeast extract peptone (YP) solution by combining 20 g/L peptone (RPI, P20240-1000.0), 10 g/L yeast extract (RPI, Y20020-1000.0), and 900 ml of double-distilled water (ddH_2_O), then autoclaved. Immediately before use, add 100 ml of sterile 20% (w/v) D-glucose (RPI, G32045-3000.0) to the YP solution to complete the YPD medium.

To prepare YPD agar plates, we used the same formulation, except that we added D-glucose (20 g/L) and agar (15 g/L; RPI, A20030-1000.0) directly to the mixture before autoclaving. We then poured the sterilized medium into 100 mm sterile Petri dishes (VWR, 25384-342) and allowed it to solidify before use.

#### Synthetic complete dropout (SCD) medium (dropout media)

We prepared uracil dropout medium (–U) by combining 5.00 g/L ammonium sulfate (Sigma-Aldrich, A4418-500G), 1.70 g/L yeast nitrogen base without amino acids or ammonium sulfate (RPI, Y20060-250), one NaOH bead pellet (ACS, BDH9292-500G), 20 g/L D-glucose (2% w/v), and 0.77 g/L CSM–Ura supplement (MP Biomedicals, 4511222). We sterilized the medium by vacuum filtration (VWR, 10040-440).

For uracil selection plates, we used the same formulation, except we added agar (15 g/L) before autoclaving to sterilize the mixture. We then poured the molten medium into 100 mm sterile Petri dishes and allowed it to solidify before use.

#### Low fluorescence SCD media (LoFo)

We prepared low-fluorescence medium by combining 8.72 g/L potassium phosphate dibasic (Sigma-Aldrich, P3786-2.5 kg), 9.76 g/L MES hydrate (RPI, M22040-500.0), 5.00 g/L ammonium sulfate (Sigma-Aldrich, A4418-500G), 1.70 g/L yeast nitrogen base—low fluorescence (without amino acids, folic acid, or riboflavin; Formedium, CYN6510), 0.79 g/L CSM Dropout Mix (complete) (MP Biomedicals, 4500022), and 20.0 g/L D-glucose (2% w/v). We adjusted the solution pH to the desired value before filter sterilization. We stored the prepared medium in the dark and allowed it to stabilize for about 3 days before using it in fluorescence-based assays.

#### 5-FoA selection plates

We prepared A 100 mM 5-FOA stock solution by dissolving 5 g of 5-fluoroorotic acid (5-FOA) (Zymo Research, F9001-5) in 50 ml of DMSO (Sigma-Aldrich, 472301-1L). We incubated the solution in a 42 °C water bath for approximately 15 min, or until the powder fully dissolved, then aliquoted it into 10 ml portions and stored it at −20 °C.

To prepare 5-FOA selection plates, we combined the following components in 900 ml of double-distilled water (ddH_2_O): 15 g agar, 20 g D-glucose, 5.00 g/L ammonium sulfate, 1.70 g yeast nitrogen base (without amino acids or ammonium sulfate), 0.79 g CSM complete mix, and 30 mg uracil (Sigma-Aldrich, U1128-100G). We added ddH_2_O to a final volume of 1 L, then autoclaved the mixture. After the medium cooled slightly, we added the 5-FOA stock solution to the molten medium, poured it into 100 mm sterile Petri dishes, and allowed it to solidify. 5-FOA plates are light sensitive and must be stored in the dark to preserve activity.

### Bacterial transformation media and agar plates

#### Luria–Bertani (LB) broth

We prepared LB broth by combining 10 g/L Bacto tryptone (RPI, T60060-500.0), 5 g/L Bacto yeast extract (RPI, Y20020-1000.0), 5 g/L sodium chloride (NaCl) (Sigma-Aldrich, 567440-1 KG), and one NaOH pellet in distilled water. We stirred the mixture until all components dissolved, adjusted to volume, and autoclaved it before use.

#### LB-ampicillin broth and plates

We prepared LB–Amp broth fresh for each experiment. We aliquoted the required volume of sterile LB broth into 15 ml (VWR, 21008-918) or 50 ml (VWR, 21008-951) conical tubes. We added a frozen stock solution of 100 mg/ml carbenicillin in 50% ethanol (AG Scientific, C-1385-25GM) to achieve a final antibiotic concentration of 0.1% (v/v), for example, 3 μl of antibiotic per 3 ml of LB broth.

We prepared LB–Amp agar plates using the same protocol as LB broth, except we added 18 g/L Bacto agar before autoclaving. After sterilization, we let the molten medium cool until warm to the touch, then added carbenicillin to a final concentration of 100 μg/ml. We then poured the medium into 100 mm sterile Petri dishes and allowed it to solidify at room temperature.

### Yeast transformation solutions

#### 1 × TE buffer

We prepared A 10 × TE buffer stock by dissolving 12.12 g/L Tris base (Sigma-Aldrich, 93352-1 kg) and 2.92 g/L EDTA (Sigma-Aldrich, EDS-100G) in distilled water. We diluted the stock 1:10 with water to a 1 × working solution containing 10 mM Tris and 1 mM EDTA.

#### LiOAc mix

We prepared a 10 × (1 M) lithium acetate (LiOAc) stock by dissolving 102 g/L LiOAc (Sigma-Aldrich, 517992-100G) in water. To prepare the LiOAc Mix, we combined 100 ml of 10 × LiOAc stock, 100 ml of 10 × TE buffer, and 800 ml of water to yield a final concentration of 10 mM Tris, 1 mM EDTA, and 100 mM LiOAc.

#### PEG mix

We prepared the PEG mixture by combining 40 g PEG 3350 (Sigma Life Sciences, 95904-1 kg-F), 10 ml 1 M LiOAc, and 10 ml 10 × TE buffer. The final solution contained 10 mM Tris, 1 mM EDTA, 100 mM LiOAc, and 40% (w/v) PEG 3350.

### Engineering yeast base strains

We used the *S. cerevisiae* strain BY4741 (MATa his3Δ1 leu2Δ0 met15Δ0 ura3Δ0) as the parental background for strain construction. We modified this strain to include two safe-locus expression cassettes, the X-2 landing pad (LP) and X-II5 landing pad (LP), previously developed in our laboratory 26, 28, 29, 31, resulting in the P22Δ strain. Using CRISPR/Cas9-mediated genome editing, we generated a companion protease strain in which the Hepatitis C virus NS3 protease was integrated into the X-2 LP (X-2: NS3) and its NS4A cofactor into the X-II5 LP (X-II5: NS4), producing the P22Δ–NS3–4A base strain. We provide complete genotypes of all strains used in this study in Dataset S3.

### Engineering of yeast strains containing chimeras of signaling proteins

We generated yeast strains harboring chimeric signaling proteins using the CRISPR-based PAM-scan system. We designed all guide plasmids and CRISPR payloads using the computational pipeline described above. We verified successful genomic integrations by PCR amplification of genomic DNA with chimera-specific primers and, in selected cases, sequenced the resulting chimeric loci for downstream applications. We detected no CRISPR editing errors in any of the sequenced constructs.

#### Mating pathway chimera screen

We integrated each mating pathway chimera into two companion yeast strains. To generate each chimera, we inserted the Hepatitis C virus NS3/4A protease recognition sequence (EDVVCCSMSY) in frame at selected codon positions in key mating pathway components. We performed integration in both the P22Δ strain, which lacks the protease, and the P22Δ-NS3-4A strain, which expresses the HCV NS3 protease from the X-2 locus (X-2: NS3) together with its NS4A cofactor from the X-II5 locus (X-II5: NS4). We then evaluated the functional activity of each resulting chimera using the assay protocol described below.

#### Yeast Ste2 and human GPCR reporter chimeras

We engineered each yeast-human GPCR chimera using a two-step CRISPR integration process. In Step 1, using the P22Δ base strain, we integrated the desired GPCR genes into the X-2 landing pad using the approach established in our previous DCyFIR studies 26, 28, 29, 31. In Step 2, the strains from Step 1 served as the base strains for chimeric integrations, in which we CRISPR-integrated the nLuc and mNeonGreen reporter genes in frame at selected codon positions within each GPCR. We subsequently evaluated the functional activity of all resulting chimeras using the assay protocol described below.

### Functional validation of PAM-scan chimeras

#### General culture conditions

We inoculated yeast P22Δ chimera strains into individual wells of a 96-deep-well block containing 1 ml of low-fluorescence (LoFo) medium (pH 6.0) and incubated overnight at 30 °C without shaking. The next day, we transferred 40 μl of saturated culture to a black 384-well microplate (Greiner, 781096), shook at 2200 rpm for 1 min, and measured the optical density (OD_600_) using a Clariostar plate reader. We diluted saturated cultures to a final OD_600_ of 0.3 in 500 to 750 μl of LoFo medium (pH 6.0) and incubated them at 30 °C for approximately 5 h, typically reaching an OD_600_ of 0.6 to 1.2.

#### Functional validation of yeast mating pathway chimeras

About 1 h before yeast cultures reached the desired optical density, prepare 384-well assay plates. We thawed a stock solution of α-factor ligand (Genescript, RP01002) from frozen and prepared it by dissolving the powdered peptide in nuclease-free water (nfH_2_O; RPI, 7732-18-5). We determined ligand concentration using a NanoDrop spectrophotometer with the Protein A_280_ protocol (“Other protein E & MW”) employing an extinction coefficient of 12,490 M^−1^ cm^−1^ and a molecular weight of 1683.96 Da. We prepared working stocks of 100 μM and 10 μM α-factor in nfH_2_O. For each yeast strain, we dispensed 4 μl of either 100 μM or 10 μM α-factor into four replicate wells (n = 4), while vehicle control wells received 4 μl nfH_2_O.

After approximately 5 h of culture growth, we measured the yeast for OD_600_ and diluted it in LoFo medium (pH 6.0) to a final OD_600_ of 0.1. We seeded 36 μl of the diluted culture into each ligand-containing well (n = 4 technical replicates), resulting in final α-factor concentrations of 10 μM or 1 μM. We sealed the plates with breathable film, shook them at 2200 rpm for 1 min, and incubated them at 30 °C for 18 to 24 h.

We measured fluorescence of the mTq2 transcriptional reporter using a Clariostar plate reader and the following settings.

• Measurement type: Fluorescence (endpoint)
• Flashes per well: 10
• Excitation: 430–10 nm
• Dichroic filter: LP 458
• Emission: 482–16 nm
• Gain: 1300
• Focal height: 6.5 mm
• Settling time: 0.5 s
• Reading direction: Bidirectional (left to right, top to bottom)

#### Functional validation of human GPCR chimeras

We evaluated the functionality of human GPCR chimeras using the GαZ DCyFIR yeast strain developed in our prior work 26, 28, 29, 31. We tested the yeast GPCR chimeras Ste2–nLuc and Ste2–mNeonGreen in the same P22Δ strain background used for protein–peptide chimeras. We applied ligands at the following final concentrations: 1 mM melatonin (Sigma-Aldrich, M5250-1G) for MTNR1A–nLuc, 1 mM adenosine (Sigma-Aldrich, A9251-25G) for A2A–nLuc, and 10 μM α-factor for Ste2–nLuc and Ste2–mNeonGreen chimeras. We incubated yeast cultures with their respective ligands for 18 to 24 h at 30 °C, then measured mTq2 reporter fluorescence using a Clariostar plate reader with the same settings as above.

In parallel, using independent yeast cultures, we confirmed the functionality of the nLuc and mNeonGreen reporter inserts. For nLuc validation, we added the Nano-Glo Luciferase Assay System (Promega) directly to the yeast culture at a final concentration of 0.2 × and measured luminescence immediately using the Clariostar (emission filter: 470–80 nm). To assess mNeonGreen activity, we recorded fluorescence on the same instrument (excitation: 430–10 nm; dichroic filter: LP 458; emission: 482–16 nm).

### Protease cleavage/protection and inhibitor rescue assays

#### Assessment of protease cleavage

We further characterized functionally validated Hepatitis C protease recognition site chimeras in two stages. In the first stage, we assessed whether the NS3/4A protease cleaved the chimeric mating pathway signaling component, as indicated by a loss or reduction in signaling activity. We prepared yeast cultures according to the general culture conditions described above, treated them with an α-factor agonist, incubated them at 30 °C for 18 to 24 h, and analyzed signaling output using the mTq2 transcriptional reporter on a Clariostar plate reader.

#### Inhibitor rescue assay

In stage two, we evaluated whether cleavage-induced signaling loss could be rescued by the Hepatitis C protease inhibitor Glecaprevir (MedChem Express, HY-17634). We conducted experiments under the same general culture conditions as above, except we used 32 μl of yeast culture (OD_600_ = 0.1) per well. Each well received 4 μl of 100 μM α-factor and 4 μl of Glecaprevir working stock at final concentrations of 1 μM, 10 μM, or 100 μM. Vehicle control wells contained 4 μl of 10% DMSO (Sigma-Aldrich, 472301-1L). We performed all conditions in technical quadruplicate (n = 4).

#### Basal signaling Gpa1 chimeras

For Gpa1 chimeras with inserted-induced basal signaling, we omitted α-factor. Wells received 4 μl of nfH_2_O in place of ligand, followed by 32 μl of yeast culture (OD_600_ = 0.1). Final glecaprevir concentrations were 1, 10, and 100 μM.

#### Preparing stock glecaprevir solutions

We reconstituted glecaprevir in 100% DMSO to 10 mM, stored it at −20 °C, and thawed it immediately before preparing working stocks, each containing 10% DMSO.

### Functional characterization of human GPCR–nLuc chimeras in HEK293T cells

#### Mammalian cell transfection

HEK293T cells were obtained from the American Type Culture Collection (ATCC, CRL-3216). The cell line was not independently authenticated by short tandem repeat profiling in this laboratory. The cell line tested negative for *mycoplasma* contamination. On the first day of experimentation, we seeded HEK293T cells at a density of 3 × 10^6^ cells per 10-cm dish in complete Dulbecco’s Modified Eagle Medium (DMEM; ThermoFisher, 11995081) and incubated at 37 °C in a humidified 5% CO_2_ atmosphere for 24 h to reach approximately 80% confluency. The next day, we transfected each dish with a total of 4 μg plasmid DNA, consisting of 2 μg of the GPCR plasmid and 2 μg of the pGloSensor-22F plasmid (Promega, GU174434). We performed transfections using the TransIT transfection reagent (Mirus Bio, MIR 5405) at a 3 μl reagent:1 μg DNA ratio, following the manufacturer’s protocol. We then incubated cells under standard culture conditions (37 °C, 5% CO_2_) for 24 h post-transfection.

#### Assay setup in 384-well plates

We harvested transfected cells by adding 1.5 ml of trypsin (Gibco) to each 10-cm dish and briefly incubating to facilitate detachment. We neutralized trypsinized cells with complete medium, collected them, and centrifuged them to pellet. We removed the supernatant and resuspended the pellet in 14 ml of fresh DMEM. Using a multichannel pipette, we plated 40 μl of cell suspension per well into white 384-well plates (Greiner, 781098) at a final density of approximately 20,000 to 40,000 cells per well, with n = 3 technical replicates per condition for the GloSensor assays and n = 4 technical replicates per condition for the nLuc assays. We incubated plates for 24 h at 37 °C and 5% CO_2_ to allow cell adherence and recovery.

#### Luciferin loading

On day 4, we aspirated the culture medium from each well using a vacuum aspirator. Each well then received 20 μl of 1 × D-luciferin solution (GoldBio, LucNA) prepared in HBSS (Gibco) supplemented with 20 mM HEPES (pH 7.4). We incubated plates at 37 °C, 5% CO_2_, for 1 h to allow luciferase substrate uptake and signal stabilization before ligand addition.

#### Assessment of melatonin–nLuc receptor chimeras

Following luciferin loading, we added 10 μl of isoproterenol (ISO; Sigma-Aldrich, I5627-5G) to achieve a final concentration of 250 nM in a total reaction volume of 40 μl. We incubated plates at room temperature (RT) for 15 min. We then performed a serial ligand dilution assay (1:3) by adding 10 μl of melatonin at various concentrations (n = 3 technical replicates) to each well, followed by an additional 15-min incubation at RT. We measured luminescence using a Clariostar plate reader under GloSensor protocol settings (measurement type: luminescence; interval: 1 s; bidirectional vertical reading, left to right, top to bottom).

#### Assessment of adenosine–nLuc receptor chimeras

For A2A–nLuc chimeras, we evaluated ligand activity using a 1:10 serial dilution assay. After luciferin loading, we added 10 μl of adenosine to each well, yielding a final well volume of 30 μl. We incubated plates for 15 min at RT before luminescence measurement using the same Clariostar GloSensor protocol as described above.

#### nLuc signaling assay

To evaluate the activity of nLuc in the human GPCR–nLuc chimeras, we diluted furimazine substrate (Promega, Nano-Glo Luciferase Assay) 100 × to 1 × working concentration. After performing the GPCR ligand assays, we measured baseline luminescence after ∼1 h to confirm minimal background signal. Then, we added 10 μl of 1 × furimazine to each well, resulting in final substrate concentrations of 0.20 × for MTNR1A–nLuc and 0.25 × for A2A–nLuc chimeras. Plates were shaken in the dark at 300 rpm for 2 min, and we immediately measured luminescence on the Clariostar using the nLuc protocol (measurement type: luminescence; interval: 1 s; emission: 482–16 nm; gain: 3000; focal height: 3 mm; bidirectional horizontal reading, left to right, top to bottom).

### Structural analysis and figure preparation

#### Structure sources and rendering

We rendered all protein structures in PyMOL (version 3.1.3.1). We obtained experimental structures from the Protein Data Bank: Ste2 (7AD3), mTq2 (6YLN), mNeonGreen (5LTP), NanoLuc (5IBO), the adenosine A2A receptor (5K2D), the melatonin MTNR1A receptor (7DB6), and the full-length (1CU1) and truncated (1NS3) forms of the HCV NS3/4A protease. For the yeast proteins lacking experimental structures, we retrieved predicted models from the AlphaFold Protein Structure Database: Gpa1 (AF-P08539-F1-model_v4), Ste4 (AF-P18851-F1-model_v4), Fus3 (AF-P16892-F1-model_v4), and Ste12 (AF-P13574-F1-model_v4). These accessions refer to version 4 of the database, the release current when we performed this analysis. We did not compute new structural models in this study. In all figures, structures serve to indicate the positions of PAM-scanned insertion sites.

### Statistics and data reporting

#### Data handling

No data were excluded from the quantitative analyses. Samples were not randomized during processing or analysis.

#### Statistical analysis

We performed all statistical analyses in GraphPad Prism version 11.0.0 (93). Figure 4 contains the only statistical comparisons reported in this study. In Figure 4*H*, we compared glecaprevir- and vehicle-treated Ste2, Fus3, mTq2, and Gpa1 chimeras by two-way ANOVA with Šídák’s multiple comparisons test. In Figure 4*J*, we compared full-length and mini-protease activity for Ste2, both for the percent change in signaling and for glecaprevir rescue, by two-way ANOVA with Šídák’s multiple comparisons test, and we made the corresponding mTq2 comparisons by unpaired *t* test. In Figure 4*K*, we compared full-length and mini-protease activity for Gpa1, both for the percent change in signaling and for glecaprevir rescue, by two-way ANOVA with Šídák’s multiple comparisons test. Asterisks denote ∗∗*p* ≤ 0.01, ∗∗∗*p* ≤ 0.001, and ∗∗∗∗*p* ≤ 0.0001.

## Data Availability

All data supporting the conclusions of this article are included within the article and its supporting information. The PAM-scanning software, including the design rules used throughout, is openly available under the MIT license at https://github.com/isomlab/pam_scanning and is distributed through Bioconda, where it installs with conda install -c bioconda pam_scanning (version 1.0.1, the version used for this study).

## Supporting information

This article contains supporting information.

## Conflict of interest

The authors declare that they have no conflicts of interest with the contents of this article.
