# Supplementary material for: Systematic mapping of chimera-permissive sites by CRISPR-guided PAM scanning
Source: J Biol Chem. 2026 Oct 8;302(12):113627. doi: 10.1016/j.jbc.2026.113627 (PMC13769575; doi:10.1016/j.jbc.2026.113627)
Supplement: Supplementary file 1 [file mmc2.pdf]

Supplemental Figures and Legends

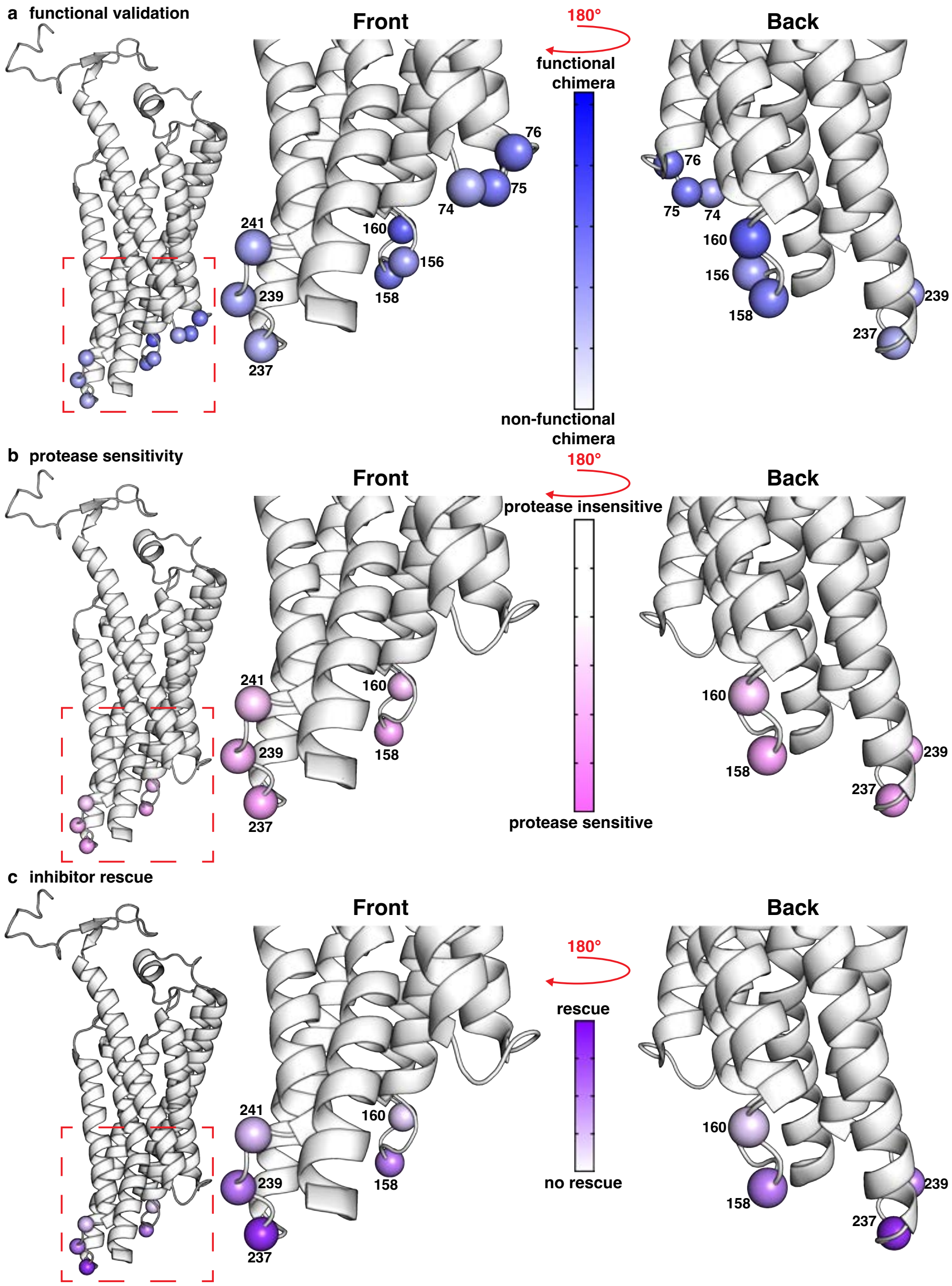

Fig. S1 | Ste2 HCV Peptide Insertion Sites.

a-c, Structural views of Ste2 (PDB: 7AD3) with PAM-scan chimeric insertion positions labeled by codon number and represented as spheres.

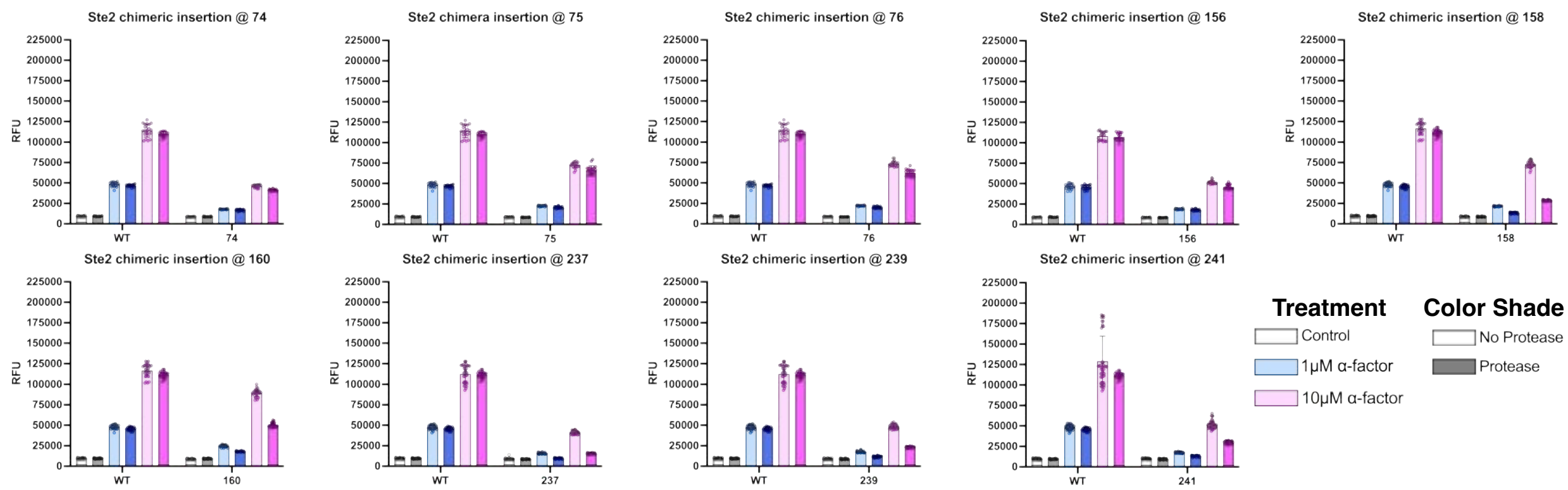

Fig. S2 | Primary functional validation data for Ste2 HCV peptide chimeras.
Pheromone pathway activation was quantified by mTq2 fluorescence and reported as relative fluorescence units (RFU). Data represent mean ± s.d. of ≥3 independent biological replicates, each with four technical replicates. Where individual points are plotted, each is a single technical replicate.

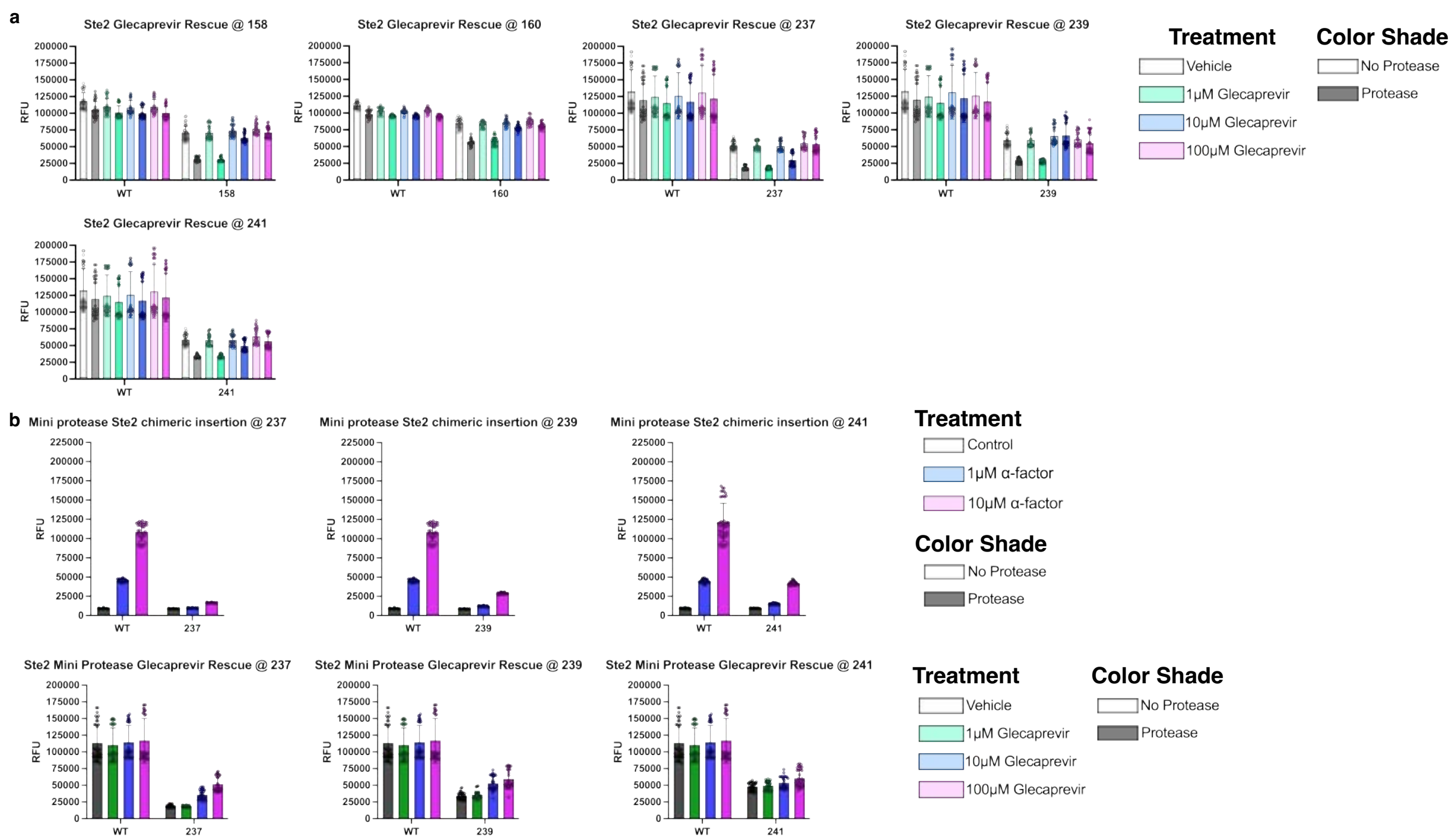

Fig. S3 | Primary functional validation, protection, and inhibitor rescue data for Ste2 HCV peptide chimeras.

a, Primary data from the inhibitor assay performed on each Ste2 site that showed reduced protease-dependent signaling.

b, Primary data from the mini-protease constructs. The α-factor assay (top) was used to derive protease activity, and the inhibitor assay (bottom) was used to calculate rescue by the inhibitor glecaprevir.

Pheromone pathway activation was quantified by mTq2 fluorescence and reported as relative fluorescence units (RFU). Data represent mean ± s.d. of ≥3 independent biological replicates, each with four technical replicates. Where individual points are plotted, each is a single technical replicate.

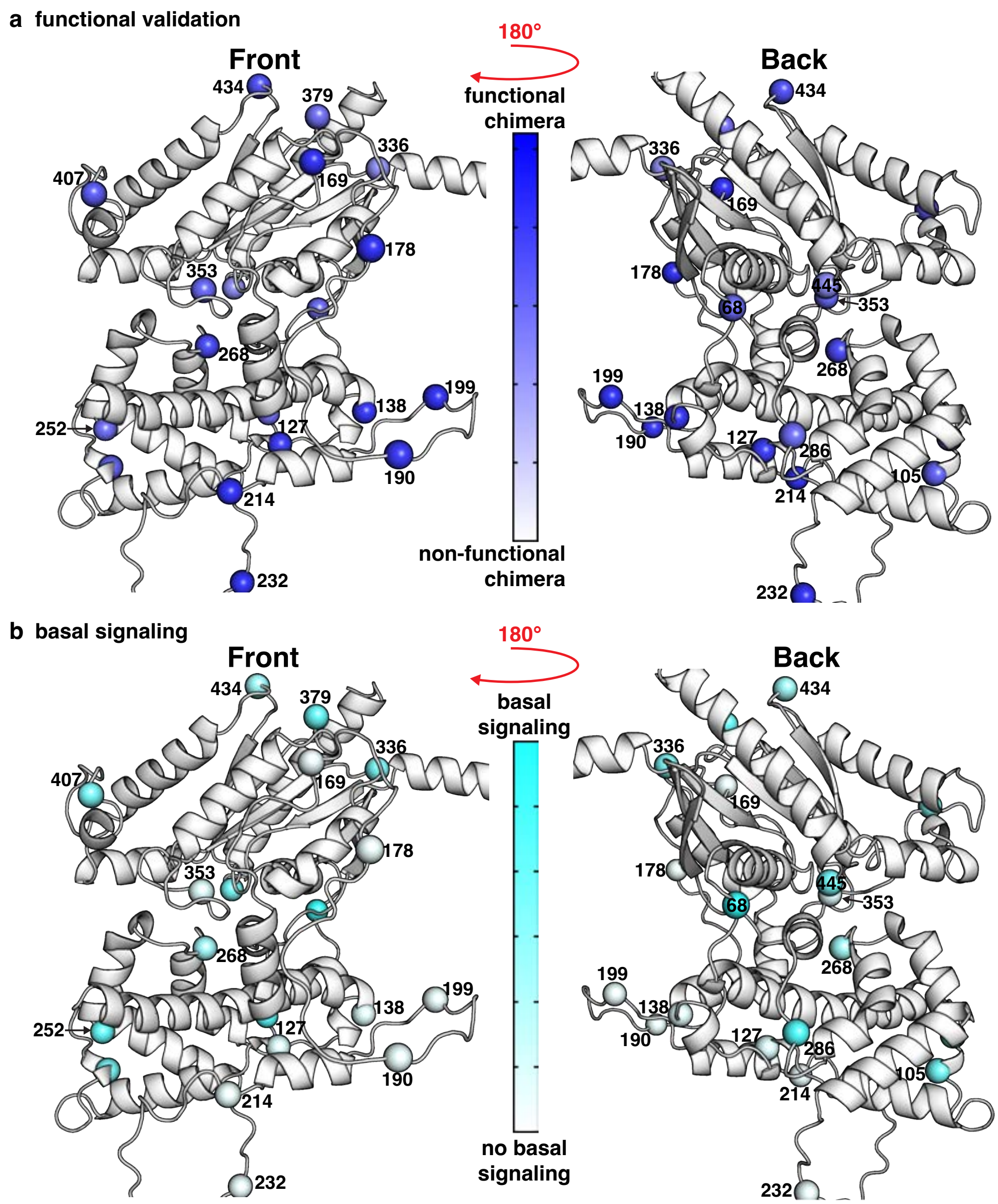

Fig. S4 | Gpa1 HCV Peptide Insertion Sites.

a-b, Structural views of AlphaFold2 model of Gpa1 (AF-P08539-F1-model_v4) with PAM-scan chimeric insertion positions labeled by codon number and represented as spheres.

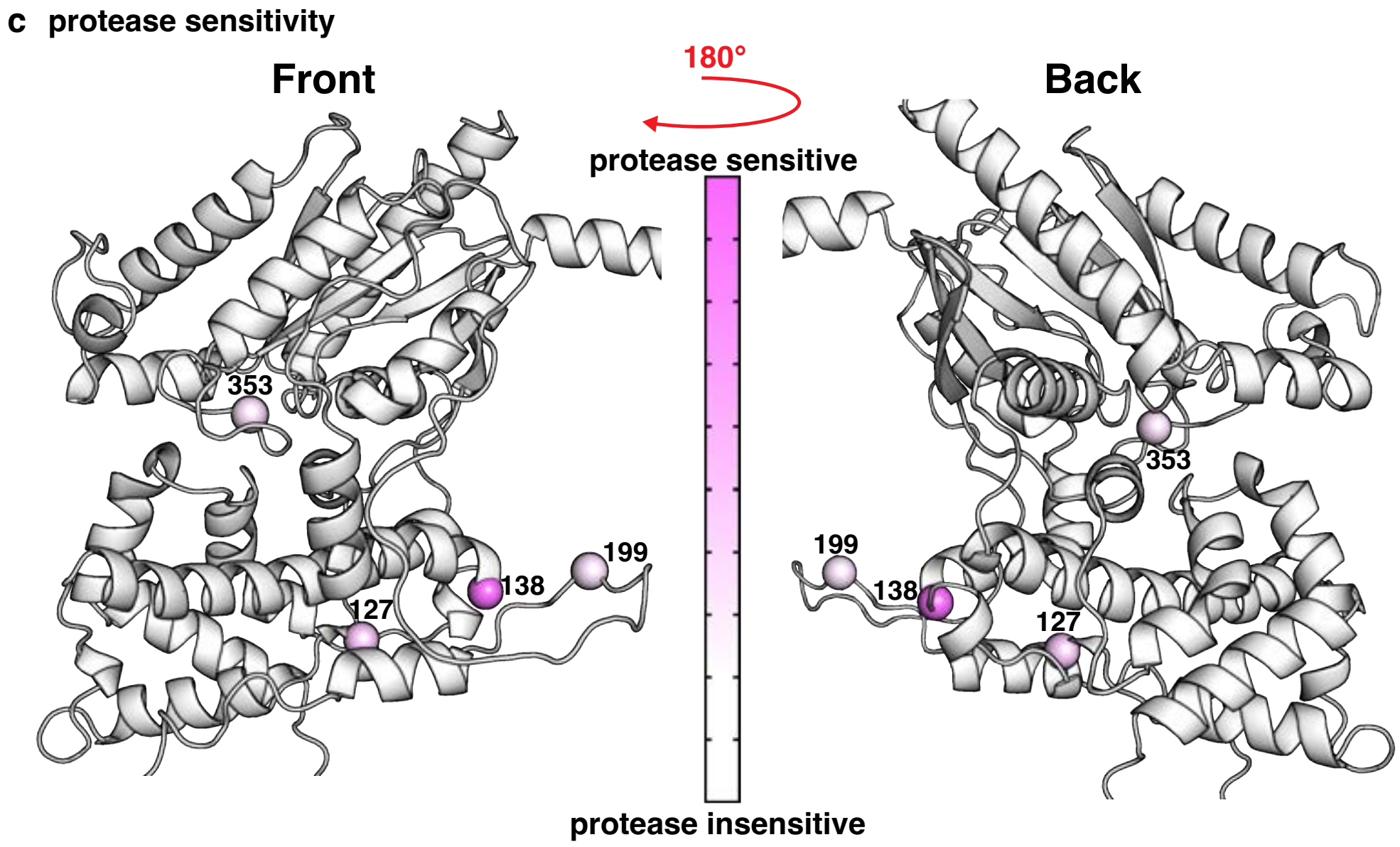

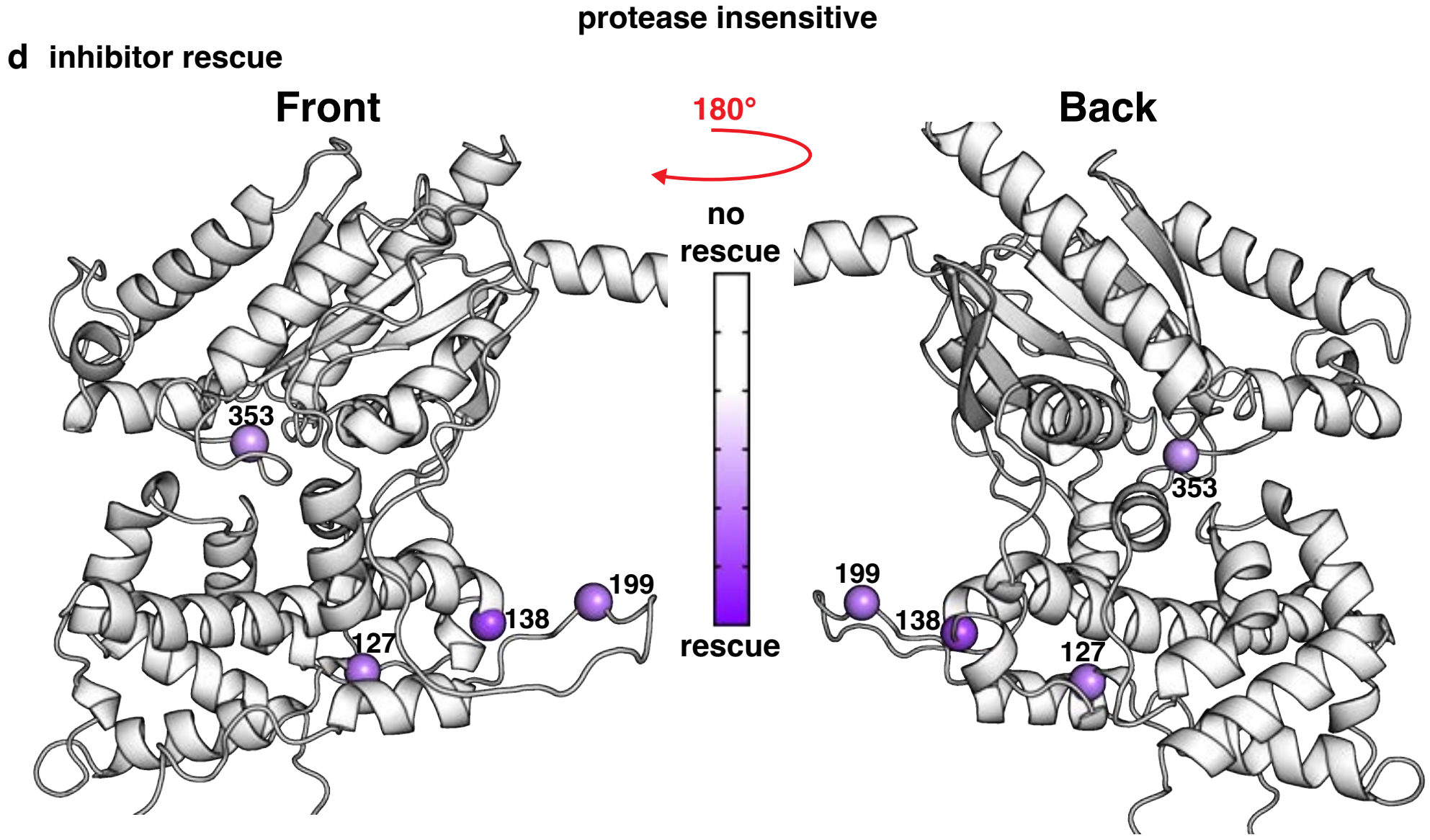

Fig. S4 (continued)

c-d, Structural views of AlphaFold2 model of Gpa1 (AF-P08539-F1-model_v4) with PAM-scan chimeric insertion positions labeled by codon number and represented as spheres.

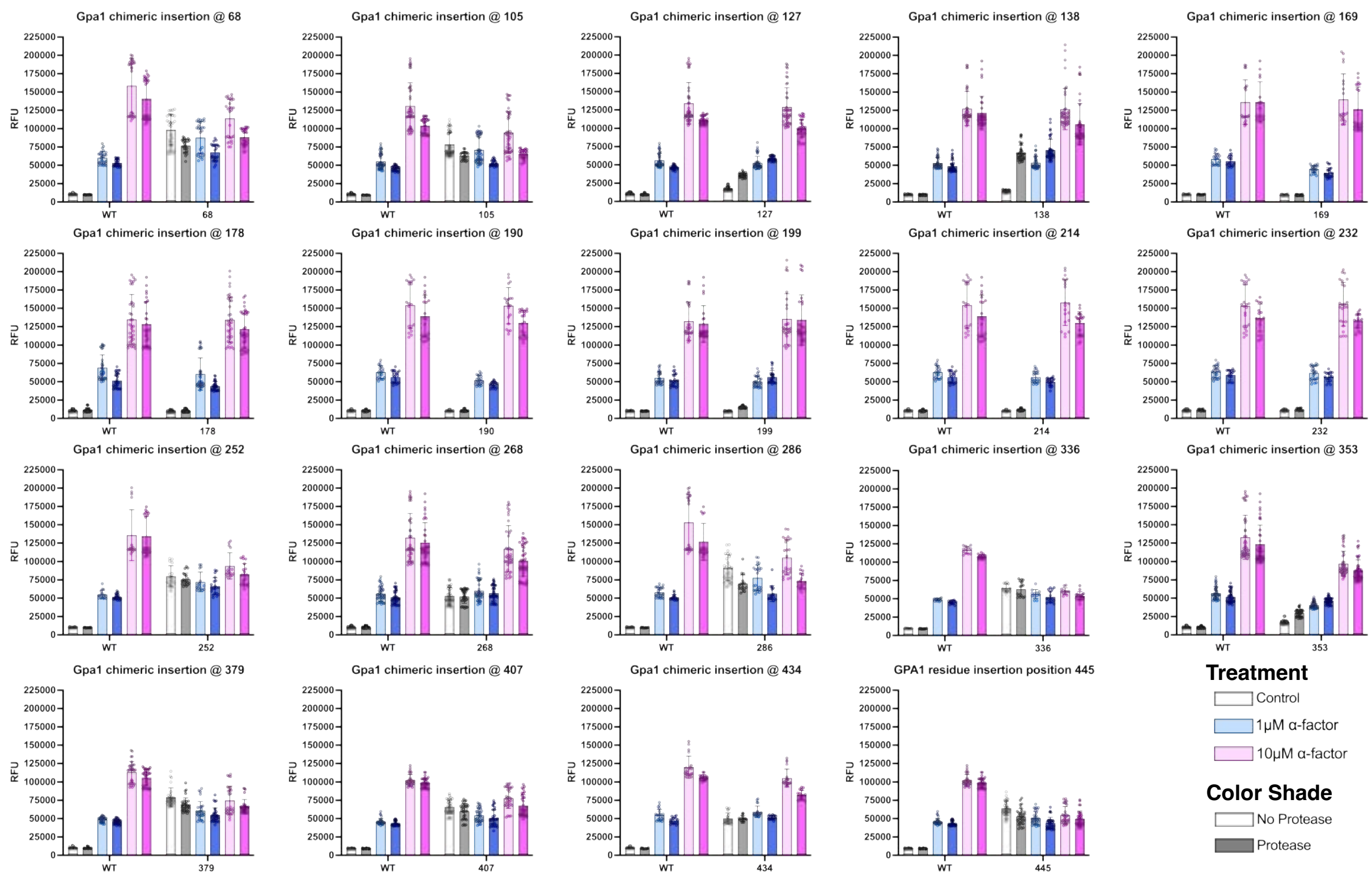

Fig. S5 | Primary functional validation data for Gpa1 HCV peptide chimeras.
Pheromone pathway activation was quantified by mTq2 fluorescence and reported as relative fluorescence units (RFU). Data represent mean ± s.d. of ≥3 independent biological replicates, each with four technical replicates. Where individual points are plotted, each is a single technical replicate.

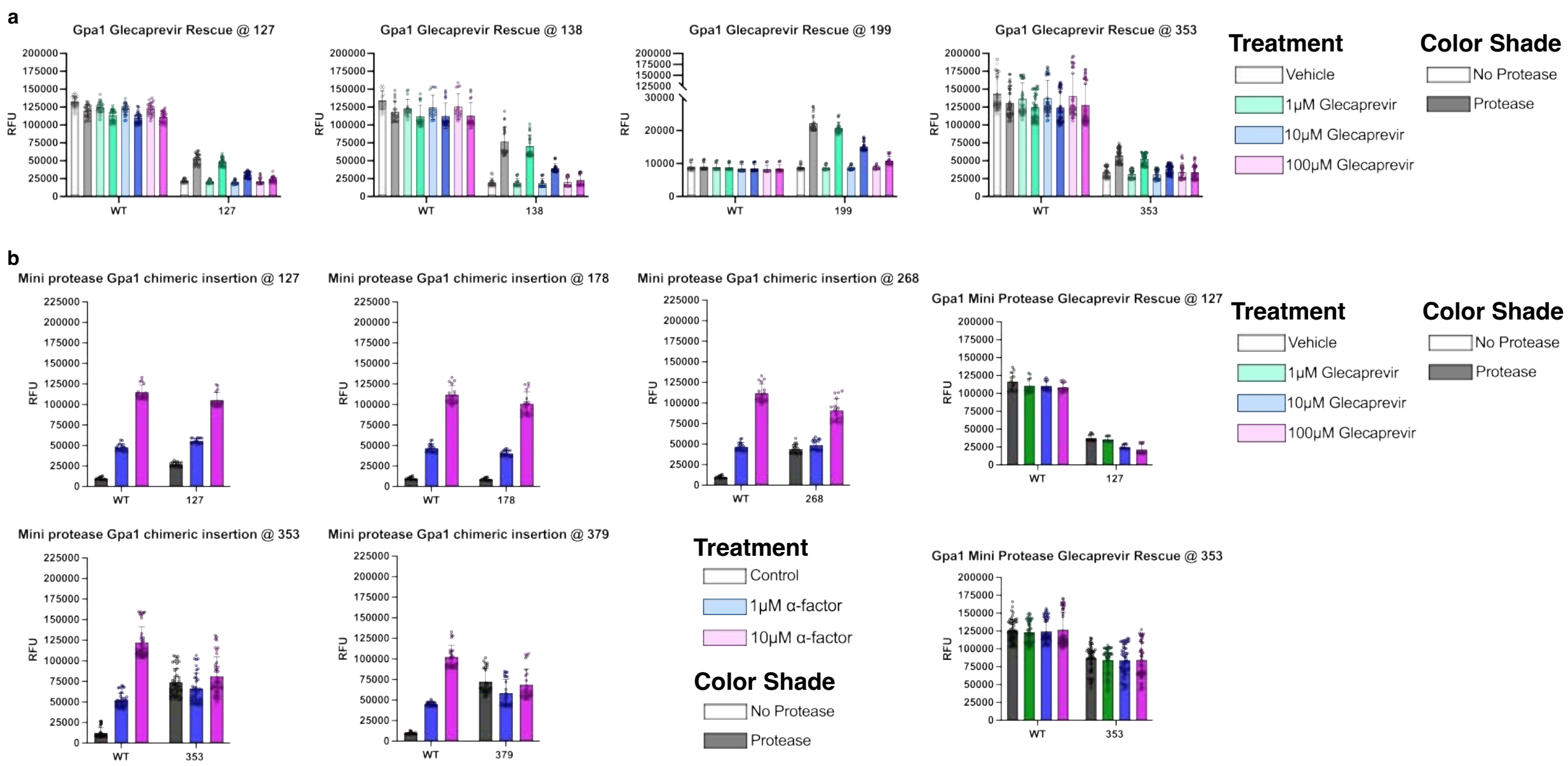

Fig. S6 | Primary functional validation, protection, and inhibitor rescue data for Gpa1 HCV peptide chimeras.

a, Primary data from the inhibitor assay performed on each Gpa1 site that showed reduced protease-dependent signaling.

b, Primary data from the mini-protease constructs. The α-factor assay (top) was used to derive protease activity, and the inhibitor assay (bottom) was used to calculate rescue by the inhibitor glecaprevir.

Pheromone pathway activation was quantified by mTq2 fluorescence and reported as relative fluorescence units (RFU). Data represent mean ± s.d. of ≥3 independent biological replicates, each with four technical replicates. Where individual points are plotted, each is a single technical replicate.

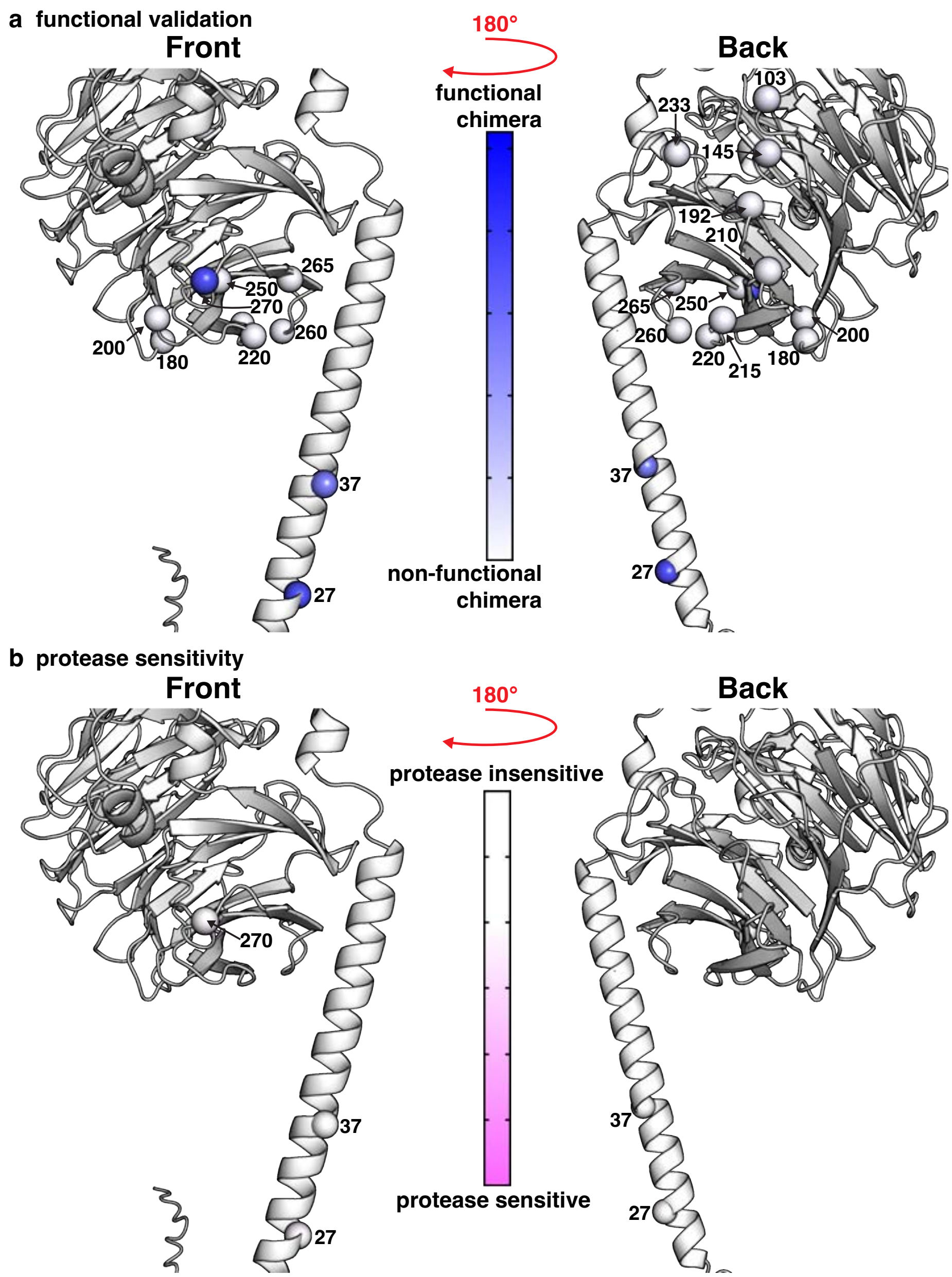

Fig. S7 | Ste4 HCV Peptide Insertion Sites.

a-b, Structural views of AlphaFold2 model of Ste4 (AF-P18851-F1-model_v4) with PAM-scan chimeric insertion positions labeled by codon number and represented as spheres.

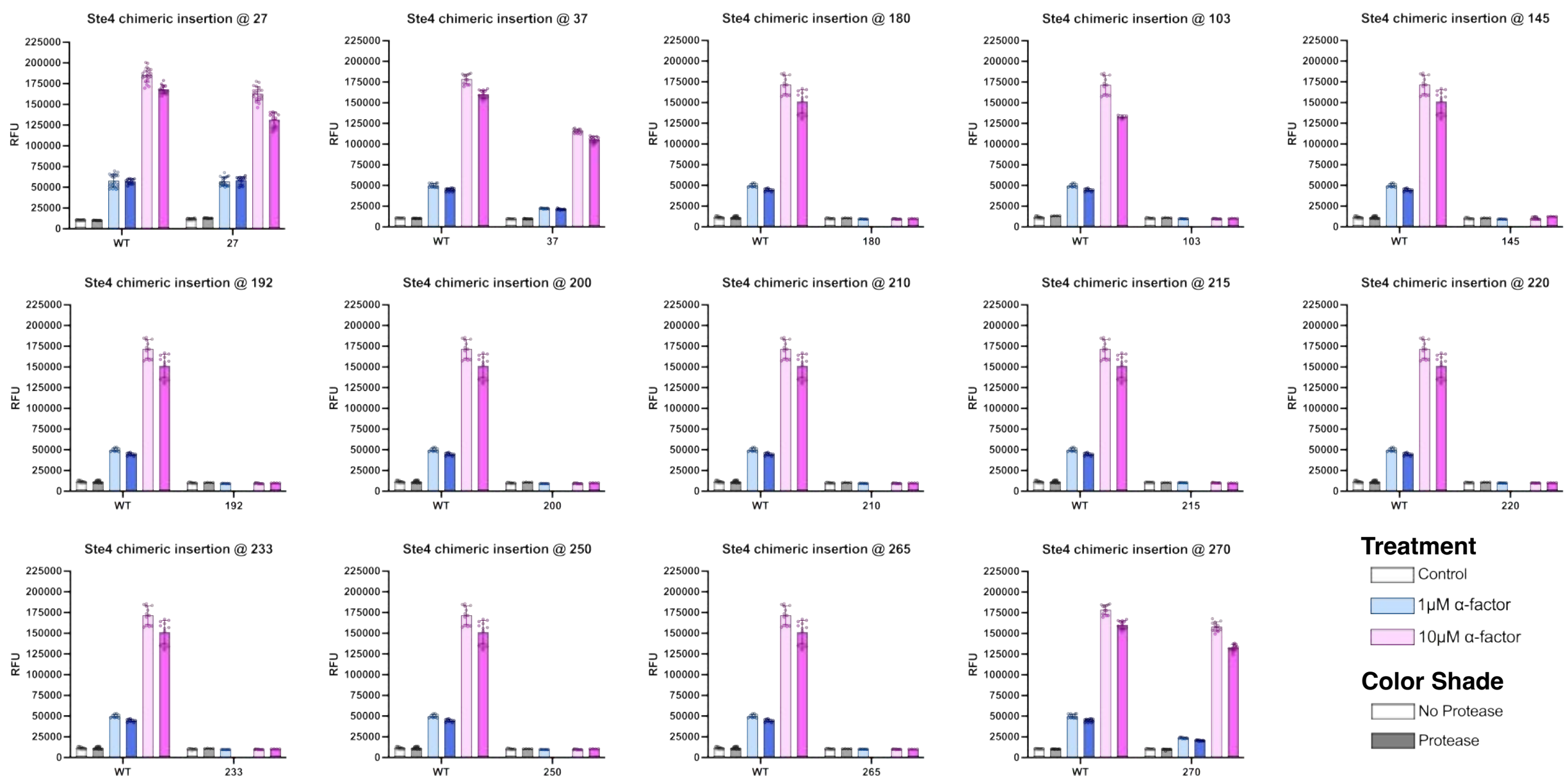

Fig. S8 | Primary functional validation data for Ste4 HCV peptide chimeras.

Pheromone pathway activation was quantified by mTq2 fluorescence and reported as relative fluorescence units (RFU). Data represent mean ± s.d. of ≥3 independent biological replicates, each with four technical replicates. Where individual points are plotted, each is a single technical replicate.

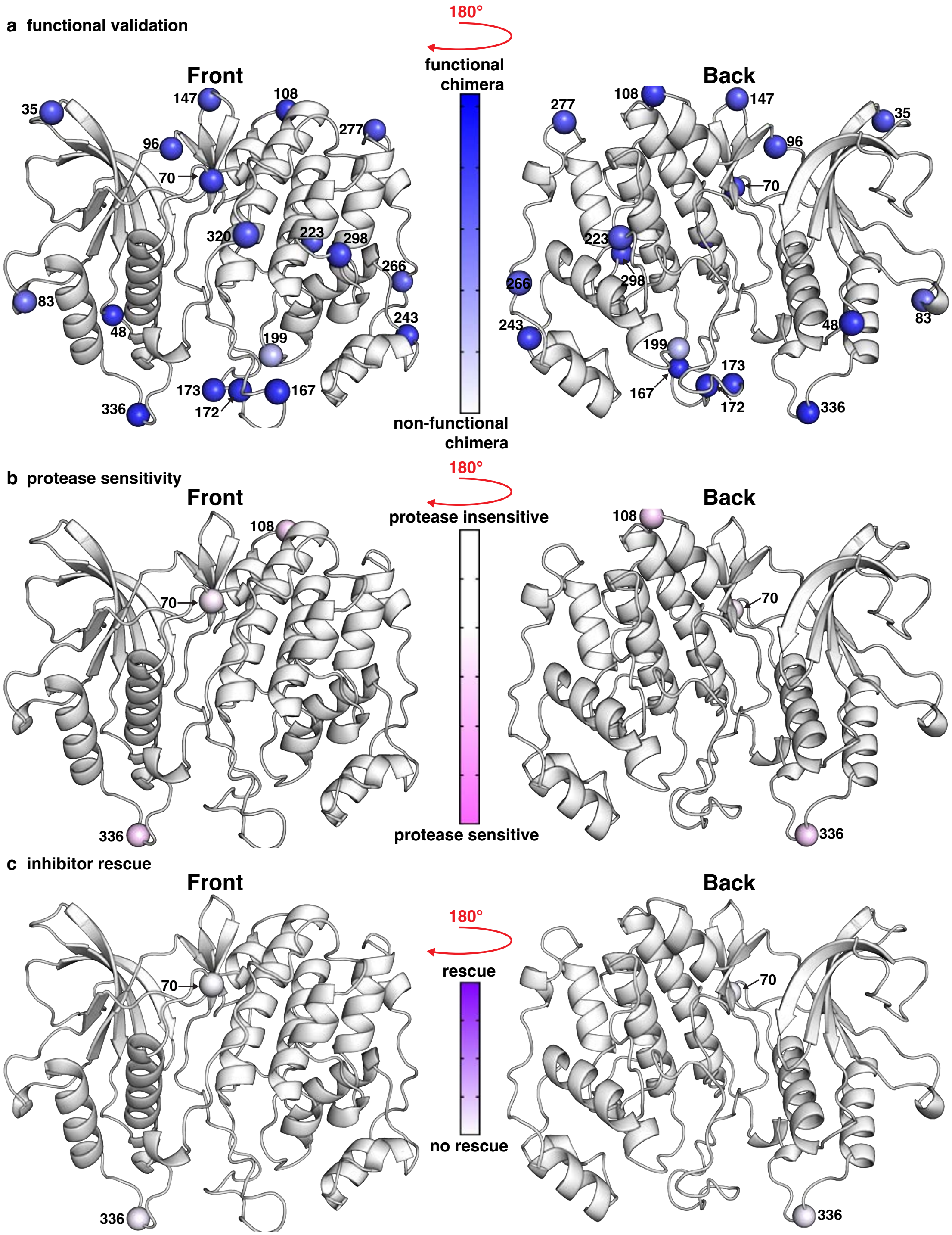

Fig. S9 | Fus3 HCV Peptide Insertion Sites.

a-c, Structural views of AlphaFold2 model of Fus3 (AF-P16892-F1-model_v4) with PAM-scan chimeric insertion positions labeled by codon number and represented as spheres.

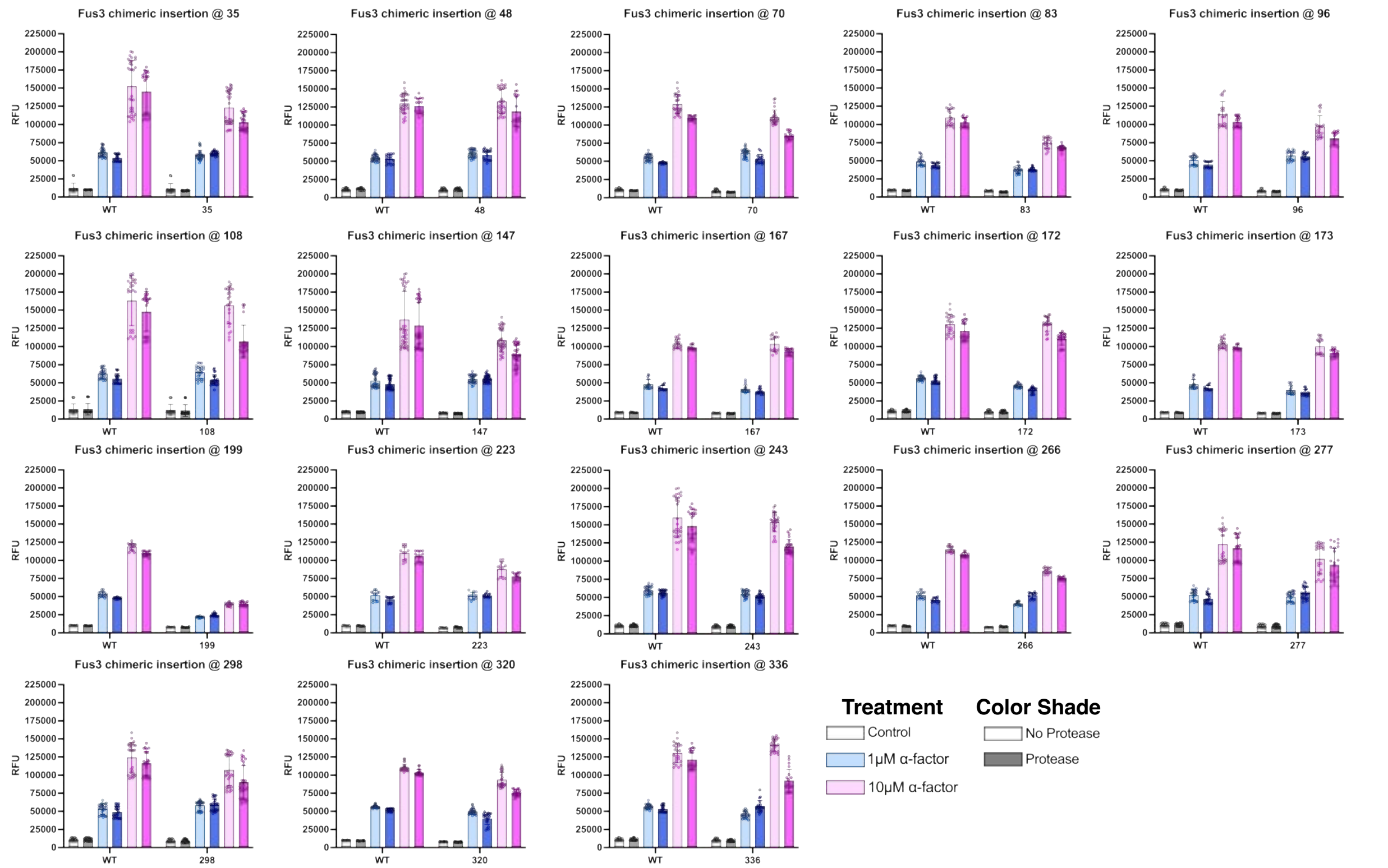

Fig. S10 | Primary functional validation data for Fus3 HCV peptide chimeras.
Pheromone pathway activation was quantified by mTq2 fluorescence and reported as relative fluorescence units (RFU). Data represent mean ± s.d. of ≥3 independent biological replicates, each with four technical replicates. Where individual points are plotted, each is a single technical replicate.

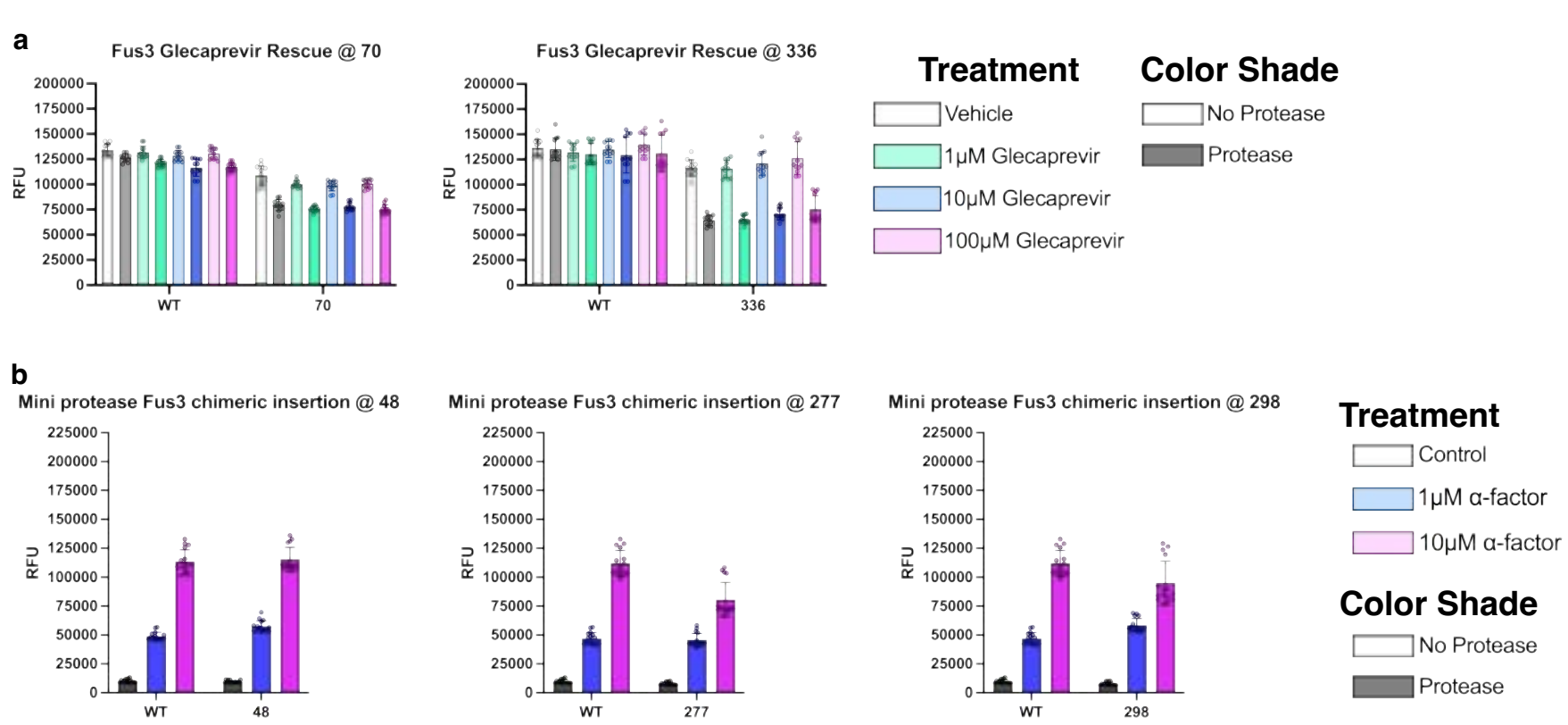

Fig. S11 | Primary functional validation, protection, and inhibitor rescue data for Fus3 HCV peptide chimeras.

a, Primary data from the inhibitor assay performed on each Fus3 site that showed reduced protease-dependent signaling.
b, Primary data from the mini-protease constructs. The α-factor assay (top) was used to derive protease activity, and the inhibitor assay (bottom) was used to calculate rescue by the inhibitor glecaprevir.

Pheromone pathway activation was quantified by mTq2 fluorescence and reported as relative fluorescence units (RFU). Data represent mean ± s.d. of ≥3 independent biological replicates, each with four technical replicates. Where individual points are plotted, each is a single technical replicate.

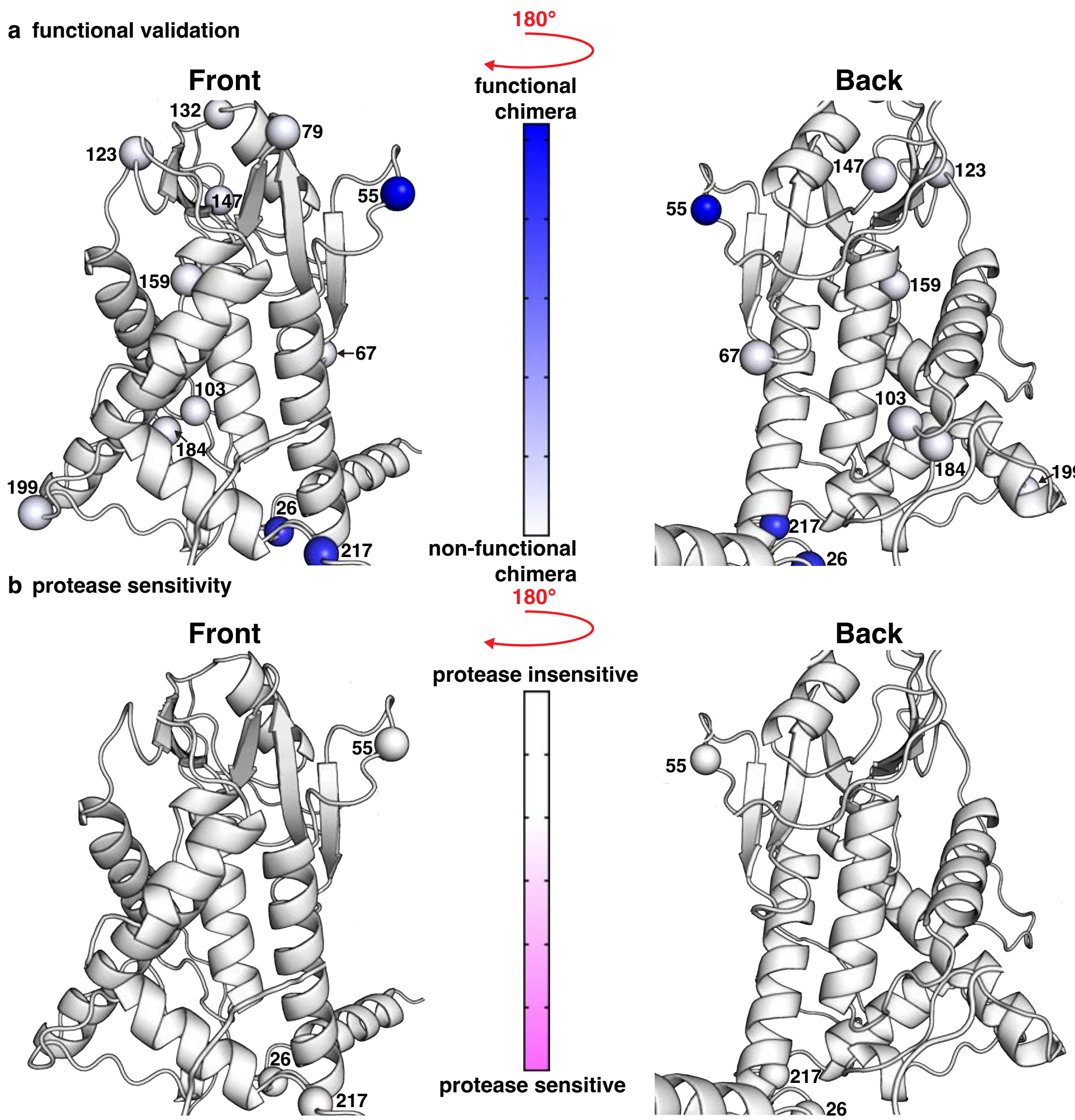

Fig. S12 | Ste12 HCV Peptide Insertion Sites.

a-b, Structural views of AlphaFold2 model of Ste12 (AF-P13574-F1-model_v4) with PAM-scan chimeric insertion positions labeled by codon number and represented as spheres. The model is cropped to display only the regions with confident structural predictions.

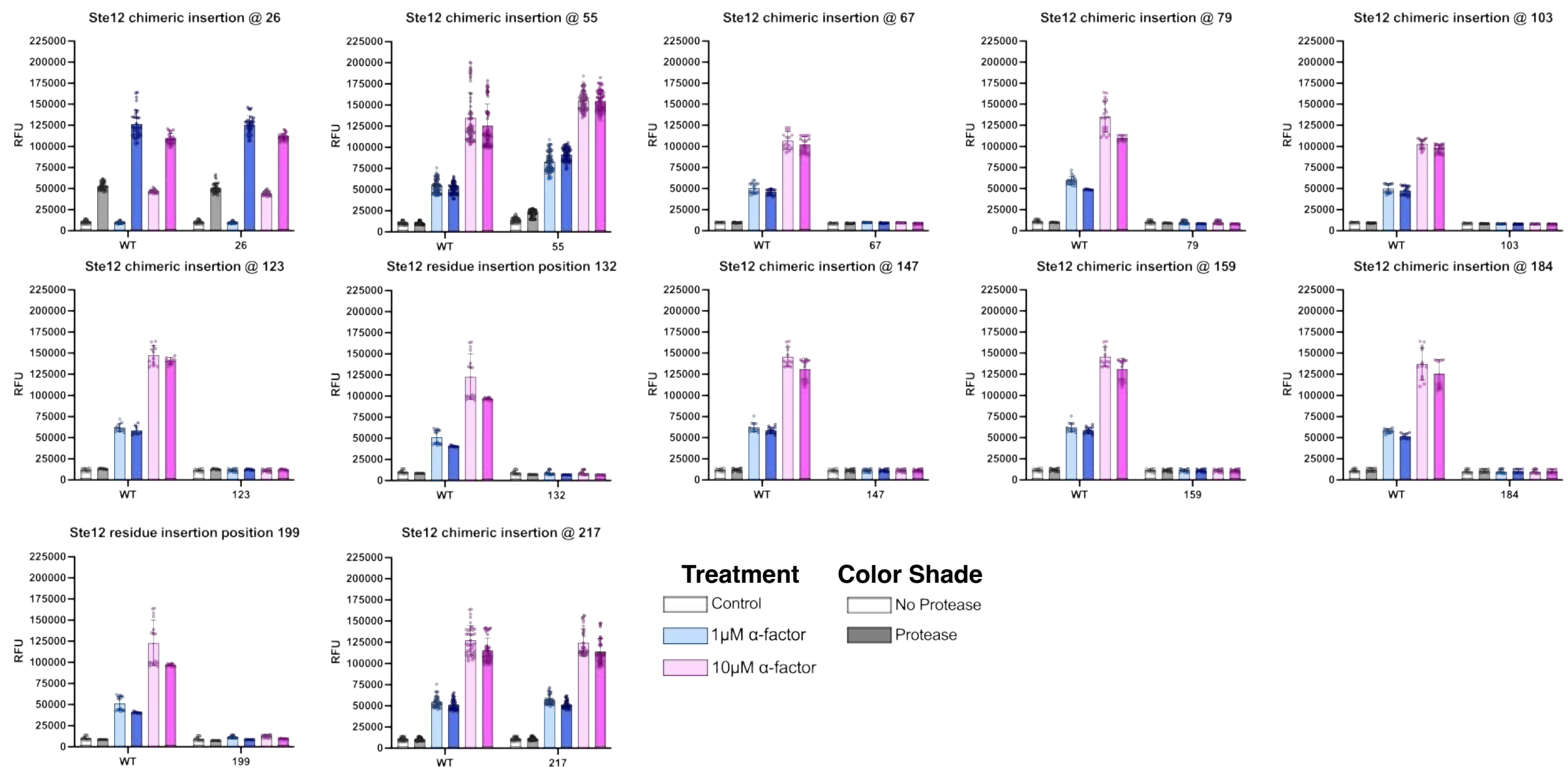

Fig. S13 | Primary functional validation data for Ste12 HCV peptide chimeras.
Pheromone pathway activation was quantified by mTq2 fluorescence and reported as relative fluorescence units (RFU). Data represent mean ± s.d. of ≥3 independent biological replicates, each with four technical replicates. Where individual points are plotted, each is a single technical replicate.

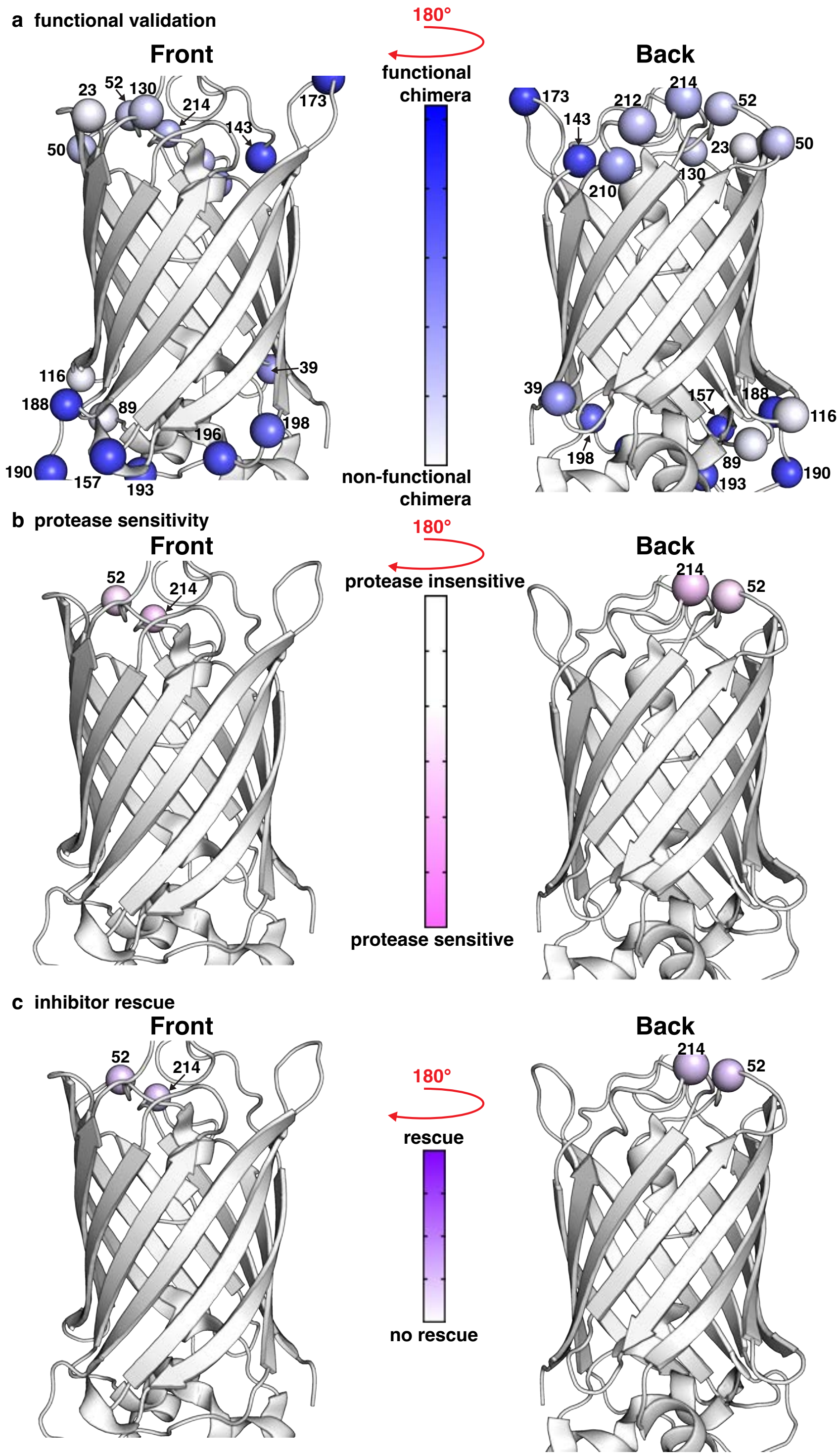

Fig. S14 | mTq2 HCV Peptide Insertion Sites.

a-c, Structural views of mTq2 (PDB: 6YLN) with PAM-scan chimeric insertion positions labeled by codon number and represented as spheres.

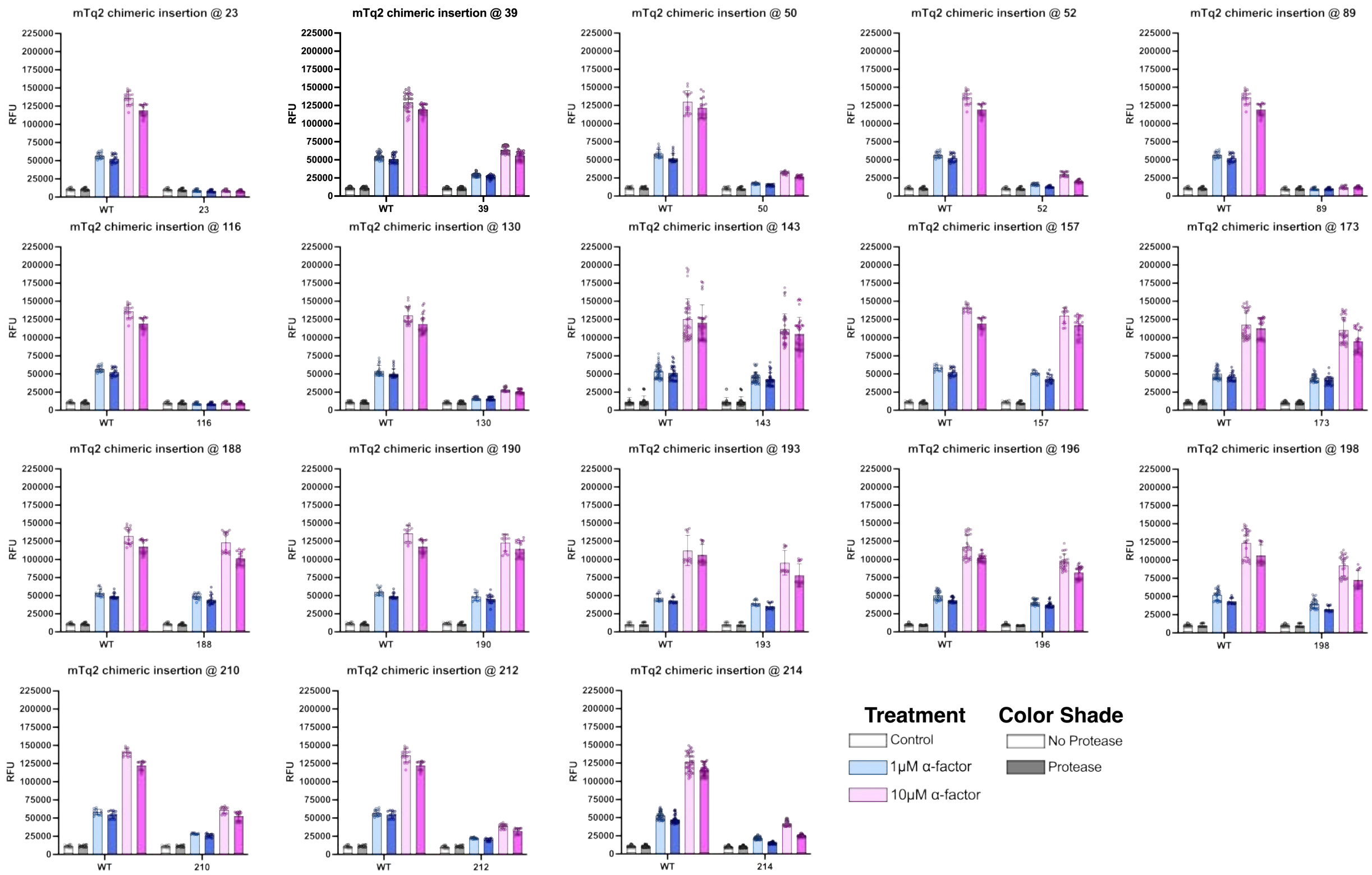

Fig. S15 | Primary functional validation data for mTq2 HCV peptide chimeras. Pheromone pathway activation was quantified by mTq2 fluorescence and reported as relative fluorescence units (RFU). Data represent mean ± s.d. of ≥3 independent biological replicates, each with four technical replicates. Where individual points are plotted, each is a single technical replicate.

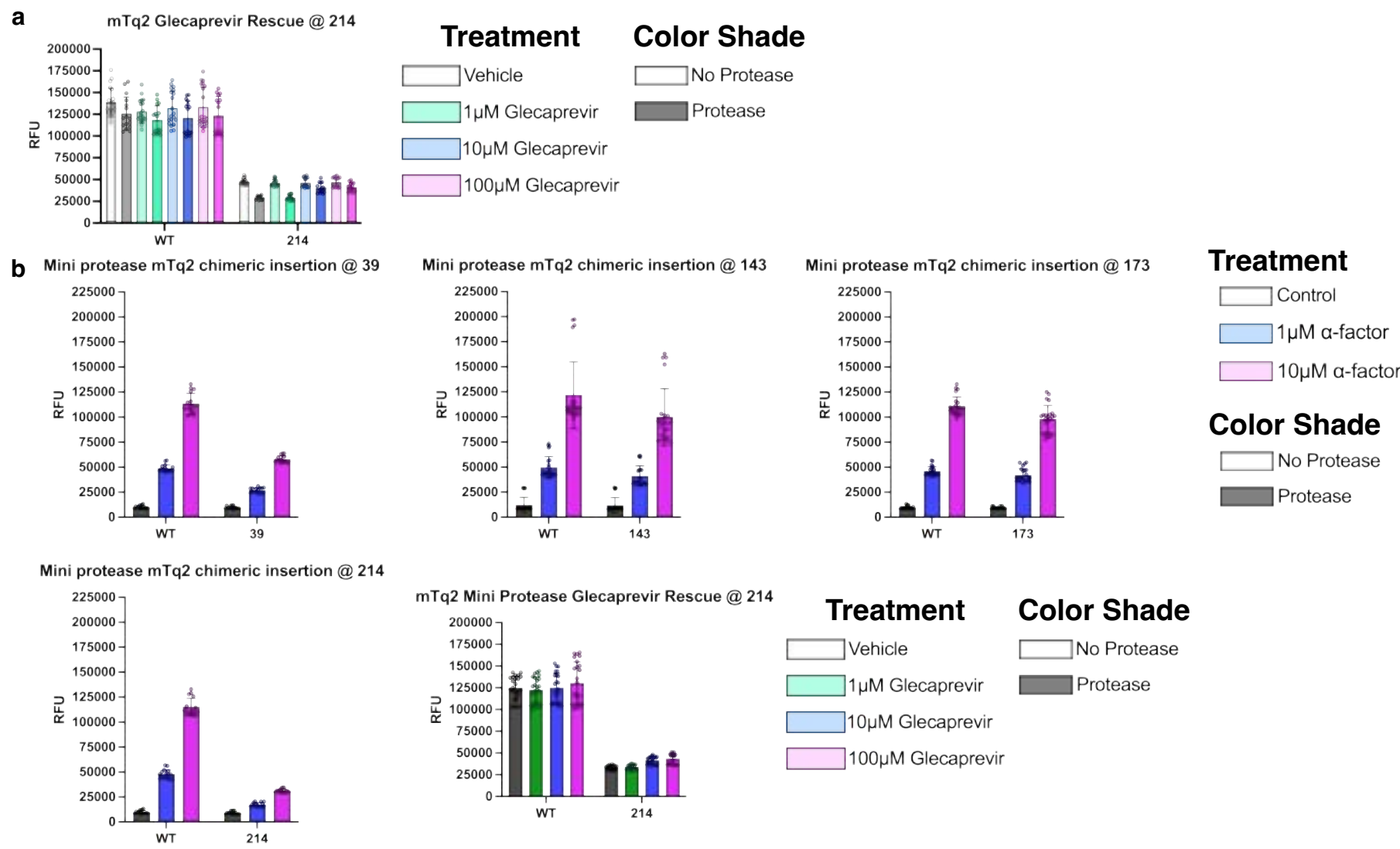

Fig. S16 | Primary functional validation, protection, and inhibitor rescue data for mTq2 HCV peptide chimeras.

a, Primary data from the inhibitor assay performed on each mTq2 site that showed reduced protease-dependent signaling.

b, Primary data from the mini-protease constructs. The α-factor assay (top) was used to derive protease activity, and the inhibitor assay (bottom) was used to calculate rescue by the inhibitor glecaprevir.

Pheromone pathway activation was quantified by mTq2 fluorescence and reported as relative fluorescence units (RFU). Data represent mean ± s.d. of ≥3 independent biological replicates, each with four technical replicates. Where individual points are plotted, each is a single technical replicate.

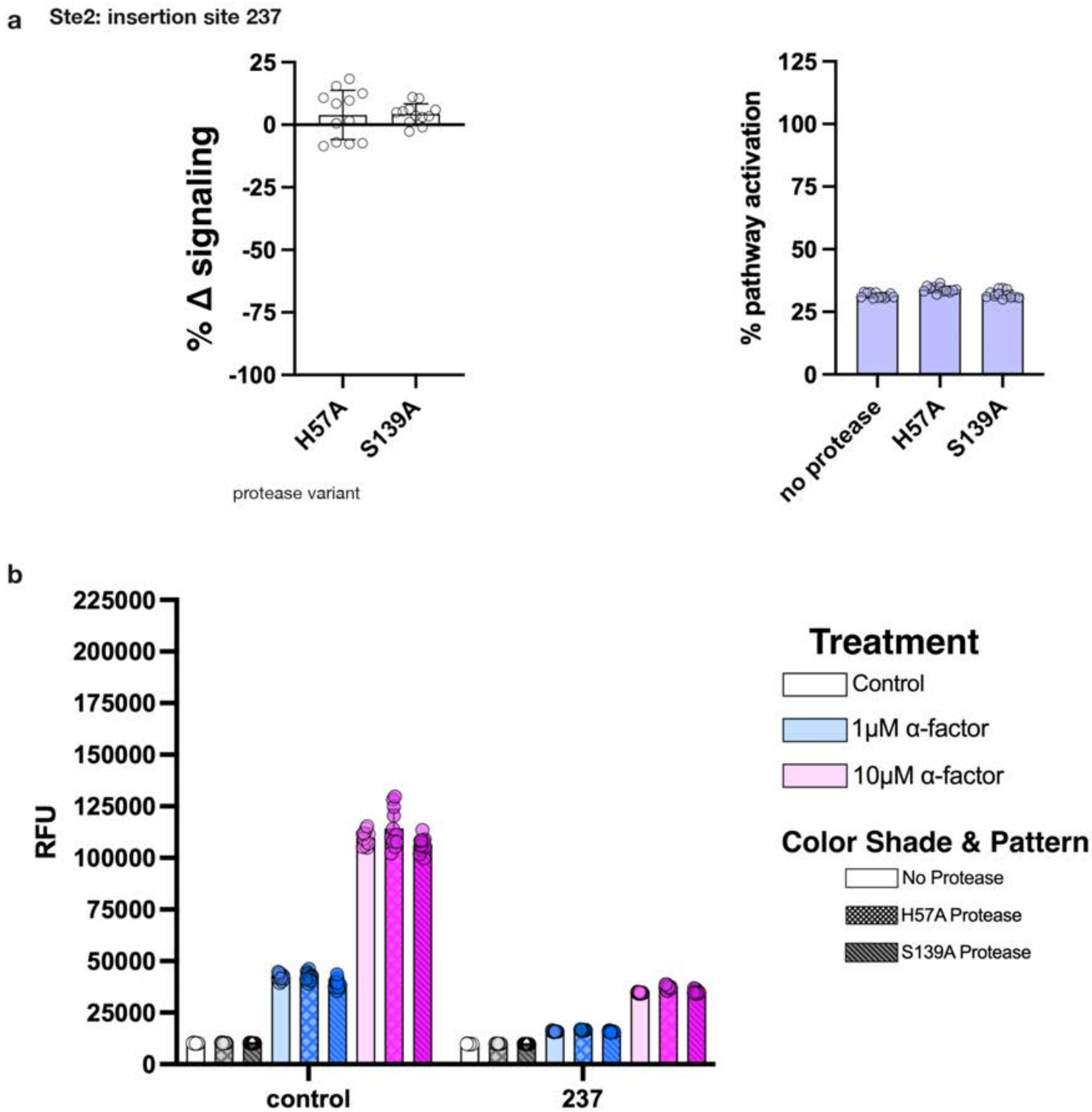

Fig. S17 | Protease-dependent changes in signaling require a catalytically active protease.

a, Protease-sensitive Ste2 insertion site 237 chimera, which carries the HCV NS3/4A recognition sequence (EDVVCCSMSY), assayed against catalytically inactive protease variants. Left, percent change in signaling for the H57A and S139A variants in the presence of 10 μM α-factor. Right, pathway activation for the same chimera expressing no protease, the H57A variant, or the S139A variant.

b, Reporter output (RFU) for the parental control strain and the site 237 chimera, untreated or stimulated with 1 μM or 10 μM α-factor, each in the presence of no protease, H57A protease, or S139A protease.

H57A and S139A each substitute a residue of the NS3 catalytic triad (His57, Asp81, Ser139) and abolish proteolytic activity. Neither inactive variant reproduced the protease-dependent change in signaling observed with the active enzyme (Fig. 4), confirming that the phenotype depends on the intended proteolytic event rather than on nonspecific processing of the inserted cassette or on protease expression itself. Cultures were normalized and incubated for 18–24 h. Data represent mean ± s.d. of ≥3 independent biological replicates, each with four technical replicates. Where individual points are plotted, each is a single technical replicate.
